# A Systematic Review and Meta-Analysis of Depression among Farming Populations Worldwide

**DOI:** 10.3390/ijerph17249376

**Published:** 2020-12-15

**Authors:** Briana N. M. Hagen, Charlotte B. Winder, Jared Wootten, Carrie K. McMullen, Andria Jones-Bitton

**Affiliations:** Department of Population Medicine, University of Guelph, Guelph, ON N1G 2W1, Canada; winderc@uoguelph.ca (C.B.W.); jwootte@uwo.ca (J.W.); cmcmulle@uoguelph.ca (C.K.M.); aqjones@uoguelph.ca (A.J.-B.)

**Keywords:** farmer, mental health, migrant farm worker, agriculture, depression, systematic review, meta-analysis

## Abstract

A systematic review and meta-analysis were conducted to determine the overall prevalence of depression among farming populations globally, and explore any heterogeneity present. Eligible studies were primary research articles published in English, which involved the collection of data for the purpose of determining the prevalence of depression among a farming population. Four relevant databases were searched in January 2019. Potential for bias was assessed using a modified Quality Assessment of Diagnostic Accuracy Studies (QUADAS) tool. From 7662 records, 72 articles were deemed relevant and had data extracted. Of these, 45 utilized the Center for Epidemiologic Studies—Depression Revised scale (CES-D/DR) to quantify depression, 42 of which were conducted in the United States (U.S.). As a result, meta-analyses were restricted to this geographic location. Substantial heterogeneity was seen in the initial whole-group analysis (*I*^2^ = 97%), and while sub-group exploration suggested a significantly higher prevalence of depression among migrant farm workers (26%, 95% CI = 21–31%) than in studies examining a non-migrant farming population (12%, 95% CI = 8–17%), substantial heterogeneity remained (*I*^2^ = 96%), indicating that the majority of between study variation was due to factors other than sampling error. Additionally, the majority of studies (81%) in migrant farm worker populations were published since 2010, while only 21% of studies in non-migrant farming populations were published in this timeframe. It is possible with recent efforts to de-stigmatize mental illness, participants in more recent studies may be more likely to self-report depressive symptoms. Hence, while it appears that migrant farmworker populations may have an elevated prevalence of depression, it is also apparent that little research in the U.S. has been done to evaluate depression among non-migrant farming populations in recent years. Perhaps a reporting bias may account for some of the difference between the two populations. A research gap also appears to exist in estimating the prevalence of depression among farming populations outside of the US. Assessment for bias at the study level revealed challenges in reporting of key study design elements, as well as potential for selection bias in the majority of studies.

## 1. Introduction

### 1.1. Rationale

Approximately one-third of the world’s population are employed through an agricultural industry, and poor mental health among farming populations may have a substantial negative impact on global economic productivity, animal health, and human health [1]. Hence, mental health among farming populations is essential for global health.

Depression is the mental illness that presents the largest mental health disease burden in higher-income countries [2]. Previous examinations of depression across farming populations have determined that depression and depressive symptoms among these groups may be elevated compared to other occupations and the general population [3,4,5]. These studies have used a variety of different metrics to assess depression and depressive symptoms among farming populations [6]. While there have been many studies, from local to national in scope, a worldwide estimate of the prevalence of depression among farming populations has not yet been calculated. Estimating the overall prevalence of depression in farming populations and identifying aspects of the population that explain differences in reported prevalence could assist in determining where additional resources should be allocated to screening for mental illness, along with intervention.

Systematic reviews provide a robust and transparent method to identify, select, and critically appraise research addressing a specific research question, which can then be quantified using meta-analysis [7]. Furthermore, assessments of heterogeneity (between study variation beyond sampling error) and exploration of heterogeneity via sub-group analysis or meta-regression can provide crucial information around the degree to which studies differ depending on the participants, methods, and outcomes that are assessed. Determining the risk for potential bias and identifying inconsistencies introduced in the methodology of individual studies can provide valuable insights to strengthen future research, leading to more precise and accurate estimates, and subsequently, more valid inferences.

### 1.2. Objectives

The objectives of this systematic review and meta-analysis were to quantify the prevalence of depression among farming populations worldwide and explore the risk of bias and heterogeneity within the included studies.

### 1.3. Protocol and Registration

The protocol for this systematic review is published in the University of Guelph repository and can be accessed at https://atrium.lib.uoguelph.ca/xmlui/bitstream/handle/10214/14603/PrevalenceDepressionFarmersSRProtocol.pdf?sequence=3&isAllowed=y. This manuscript was prepared following the Preferred Reporting Items for Systematic Reviews and Meta Analyses (PRISMA) reporting guidelines [8].

## 2. Materials and Methods

### 2.1. Eligibility Criteria

Studies were eligible for inclusion in the review if they were primary research studies available in English, with data collected for the purpose of examining the prevalence of depression within a farming population.

### 2.2. Information Sources

Electronic searches were conducted using the following databases: PubMed (via NCBI); Agricola (via Proquest); Medline (via Ovid); and Web of Science (via ProQuest). There were no date restrictions in the search aside from those of the databases themselves. The search included records published prior to 1 January 2019. To ensure completeness, results of the search were cross-referenced with the results of a literature search conducted in a previous scoping review, to describe and characterize the literature examining mental health among farming populations, which included articles published before 1 January 2019 [6].

### 2.3. Search

The search string utilized was (“mental health” OR “mental illness” OR “anxiety OR “depress*” OR “occupational stress”) AND (farm* OR agricultur*). Search terms were designed to maximize sensitivity and were informed by the prior scoping review [6]. Records were downloaded from databases using EndNote (Clarivate Analytics, Philadelphia, PA, USA) and then uploaded to DistillerSR^®^ (Evidence Partners Inc., Ottawa, ON, Canada).

### 2.4. Study Selection

Study selection, data extraction, and risk of bias were facilitated using DistillerSR^®^.

#### 2.4.1. Title and Abstract Selection

Screening was conducted by B.N.M.H., J.W., C.K.M., and C.B.W. The first 100 records were used to pre-test the title/abstract screening form between all reviewers to ensure clarity of the screening questions and consistency in classification among the reviewers. Following pre-testing, each title and abstract were assessed independently by two of the above reviewers using the following questions: (1) Is the study available in English? (2) Is the article a primary study? (including the use of census data); and (3) Does the study examine depression in a farming population? Response options for these questions included “yes”, “no”, and “unclear”. Records that received a “yes” or “unclear” response to all three questions were included in the full-text screening; records that received “no” to any of the questions were excluded. Conflicts arising between the reviewers during the title and abstract screening were resolved by consensus or mediation by B.N.H.M. or C.B.W., if needed.

#### 2.4.2. Full-Text Selection

Full-text versions were obtained for all included records that passed through the title and abstract screening. The full text screening form was pre-tested with 10 articles by all reviewers, after which articles were screened independently in duplicate. Questions used to assess eligibility of an article at the full-text screening level were (1) Is the full-text article available? (2) Is the study available in English? (3) Is the article a primary study? (including the use of census data); and (4) Does the study attempt to quantify depression in a farming population? Responses were “yes” or “no”, and records were excluded if the reviewers did not answer “yes” to each of the questions in the full-text screening. Conflicts were resolved through consensus with mediation by B.N.M.H. or C.B.W., if needed. Reasons for exclusion at full-text were recorded.

### 2.5. Data Collection

#### 2.5.1. Process

All reviewers were trained on the use of data extraction forms using the first 10 included records to ensure clarity of the form and consistency among reviewers. Following pre-testing, data extraction was done independently in duplicate, with conflicts resolved by consensus or mediation by B.N.M.H. or C.B.W, if needed.

#### 2.5.2. Items

The following data items were used to collect information pertinent to the research objectives: year of publication, year of conduct, target population, gender distribution of the sample, size of the sample, participant selection criteria, study design, and details of the outcome assessment (including if a validated scale was used to assess depression).

### 2.6. Risk of Bias within Studies

Risk of bias was assessed in all eligible studies using a previously modified version of the Quality Assessment of Diagnostic Accuracy Studies (QUADAS) tool [9], with items adapted to suit this body of literature. The QUADAS tool questions were modified in order to assess the risk of bias in observational studies quantifying depression, and included participant selection, methodology, and examination of depression in farming populations. The risk of bias form used is available as Supplementary Materials (Table S1. Risk of bias form).

### 2.7. Summary Measures

For this review, binary (i.e., data proportioned into “depressed” and “not depressed”) and continuous data (i.e., depression measured on a continuous scale) were both extracted if available. To be included, continuous data needed to also report a variance metric (e.g., standard deviation). Raw (i.e., unadjusted) and adjusted data were extracted, with priority placed on raw data.

### 2.8. Synthesis of Results

Meta-analyses were conducted for studies estimating the prevalence of depression using a validated scale. Meta-analyses were conducted in R 3.3.3 (R Foundation for Statistical Computing, Vienna, Austria), using RStudio version 1.2.5019 “Elderflower” (RStudio Inc., Boston, MA, USA), with the “meta’ package [10]. A random effects approach with the inverse variance method to weight studies was used [11]. Proportion estimates from individual studies were transformed using the Freeman–Tukey arcsine transformation to stabilize the variance [12]. Heterogeneity was assessed using the *I*^2^ statistic with a value >50% indicating substantial heterogeneity [11]. A forest plot was used to describe the prevalence of depression across articles [10].

### 2.9. Additional Analyses

Subgroup analyses were planned for population type (farmers, migrant-farmworkers, and permanent farm-workers) and type of validated scaled used, to further examine the potential heterogeneity.

## 3. Results

### 3.1. Study Selection

**Figure 1:**
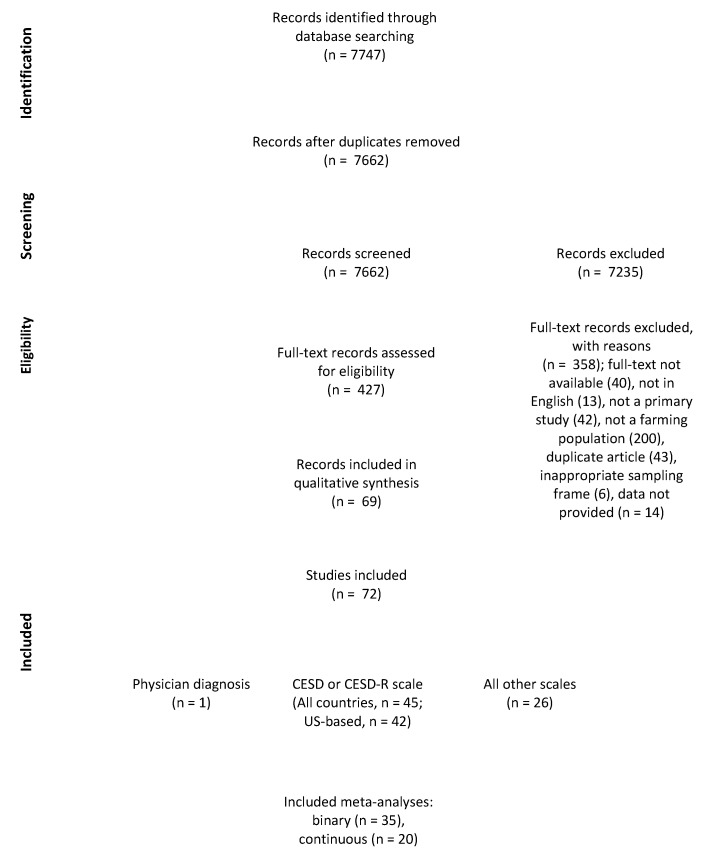
Flow chart reporting the studies included in the systematic review and meta-analysis, determining the prevalence of depression among farming populations.

The selection of studies for inclusion in the systematic review is summarized in Figure 1. From 7662 initially identified records, 427 were assessed at full-text screening, with 69 records containing 72 studies ultimately deemed eligible for data extraction. Records excluded at this stage, including reasons for exclusions, are available as Supplemental File S1 (Material S1. Full-text exclusions). Six articles collected data on depression in a farming population, but as their primary objective was not to quantify depression, these were excluded to avoid additional bias. For example, one study sampled from a hospital population of farmers with COPD, where the prevalence of depression may not reflect that of the non-migrant farming population [13].

Of the 72 studies from which data were extracted, the Centre for Epidemiologic Studies Depression scale (CES-D) (n = 44) or Centre for Epidemiologic Studies Depression Scale—Revised (CES-DR) scale (n = 1) were the most common self-reported scales used to quantify depression. One study utilized a physician’s diagnosis of depression, while the remaining 36 studies utilized other self-reporting depression scales. These included Beck’s Depression Inventory (BDI) (n = 6), Patient Health Questionnaire (PHQ) (n = 6), Hospital Anxiety and Depression Scale (HADS) (n = 4), Depression Anxiety Stress Scale (DASS) (n = 2), Geriatric Depression Scale (GDS) (n = 2), Brief Symptom Inventory (BSI) (n = 1), Composite International Diagnostic Interview (CIDI) (n = 1), Clinical Interview Schedule-Revised (CIS-R) (n = 1), Hamilton Depression Rating Scale (HAM-D) (n = 1), Montgomery–Asberg Depression Rating Scale (MADRS) (n = 1), and Short Depression Happiness Scale (SDHS) (n = 1). For scales other than CES-D/DR, data were reported as a binary (i.e., classified as having depression or not) outcome alone (n = 18), a continuous outcome alone (e.g., mean scale score and standard deviation) (n = 3), or as both a binary and continuous metric (n = 5).

Of the 45 studies utilizing the CES-D or CES-DR scales, 38 studies reported a binary outcome, and 21 reported a continuous outcome. Fourteen of these studies reported both metrics. Based on the small number of studies per scale types other than CES-D/DR, further synthesis was restricted to the studies utilizing the latter scale. Of these studies, the majority were conducted in the US (n = 42), with three conducted elsewhere (Indonesia [14], Thailand [5], and Tanzania [15]). As a result, a post-hoc decision was made to restrict the meta-analysis to the 42 studies conducted in the U.S.

### 3.2. Study Characteristics

The characteristics of the 42 studies included in the meta-analyses are provided in Table 1. Year of conduct was reported in 35 studies, with 11 (31.4%) conducted prior to 2000, 13 (37.1%) conducted between 2001 and 2010, and 11 (31.4%) conducted after 2010. Twenty-six (62%) were conducted specifically in a population of migrant farmworkers, with the remainder conducted in a non-migrant farming population. The study population was men in 13 studies (31%), women in 3 studies (7%), and a population of male and female genders in 26 studies (62%). Of studies with both male and female genders, 25/26 reported a gender proportion, with a range in the study sample from 1 to 58% female. One study additionally reported that an option for a non-binary gender was also given, although none were reported in the sample.

### 3.3. Risk of Bias

Data related to the risk of bias assessment are reported in Table 2. Half of the (21/42) included studies used a random sampling technique, while the other 50% used convenience sampling. Almost all of the studies (41/42) explicitly stated the target population, described the study population, reported the final sample size, and used the same mode of data collection for all participants within a study. Half of the studies (21/42) reported the number of participants classified as having depression as a number rather than a percentage of their sample. Over half of the studies (23/42; 54.8%) did not report a response rate. Four studies included a description of non-responders [16,17,18,19], three described the participant’s reason for refusal [17,18,19], and one study provided an analysis comparing the descriptive statistics between responders and non-responders [16].

### 3.4. Results of Individual Studies and Synthesis of Results

#### 3.4.1. Binary Outcome Data

**Figure 2:**
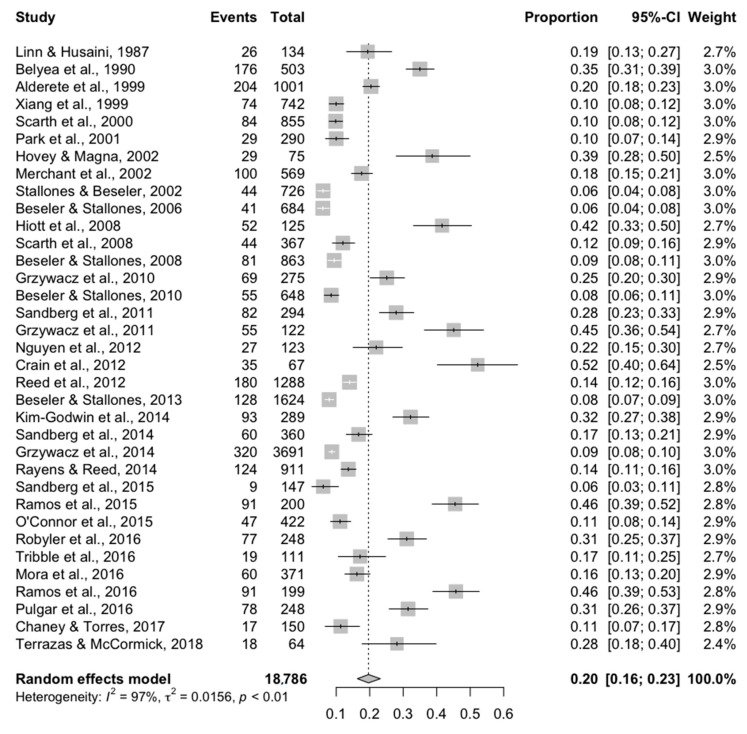
Overall meta-analysis for the 35 studies conducted in the United States reporting a binary outcome (yes/no) using the CES-D/DR scale to measure depression in farming populations.

Results of the 35 studies reporting a binary outcome using the CES-D/DR scale included in the meta-analysis are summarized in Figure 2. The overall prevalence of depression was 20% (95% CI = 16–23%), with a large degree of heterogeneity (*I*^2^ = 0.97), indicating that the majority of between-study variation in the point estimate is likely due to factors other than sampling variation.

#### 3.4.2. Continuous Outcome Data

**Figure 3:**
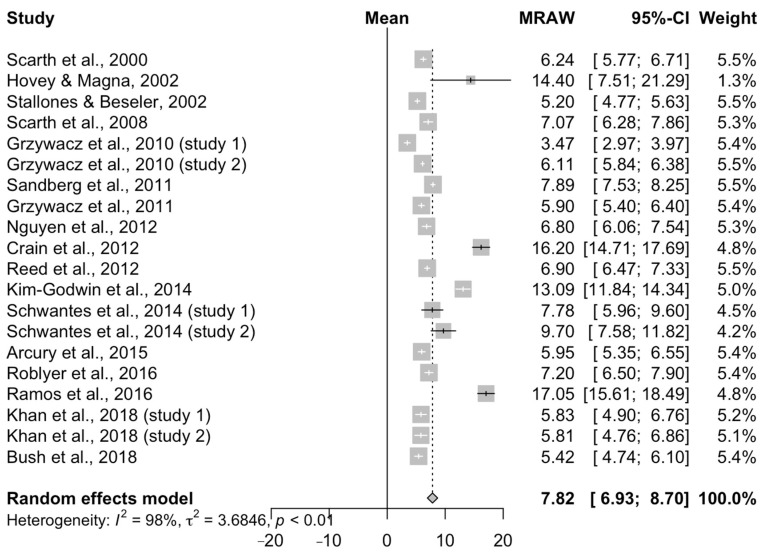
Overall meta-analysis for the 20 studies conducted in the United States reporting a continuous outcome (raw mean score, MRAW) using the CES-D/DR scale to measure depression in farming populations.

Results of the 20 studies reporting a continuous outcome (mean score) using the CES-D/DR scale included in the meta-analysis are summarized in Figure 3. The overall mean scale score was 7.82 (95% CI = 6.93–8.70), with a large degree of heterogeneity (*I*^2^ = 0.98), which also indicated that the majority of the between-study variation seen in the mean score is likely attributable to factors other than random sampling error.

### 3.5. Additional Analyses

Subgroup analysis by scale type was not possible, as although several different scales were used, there were not enough scales reporting the same outcome metric (i.e., binary or continuous) to be combined in a single sub-group.

**Figure 4:**
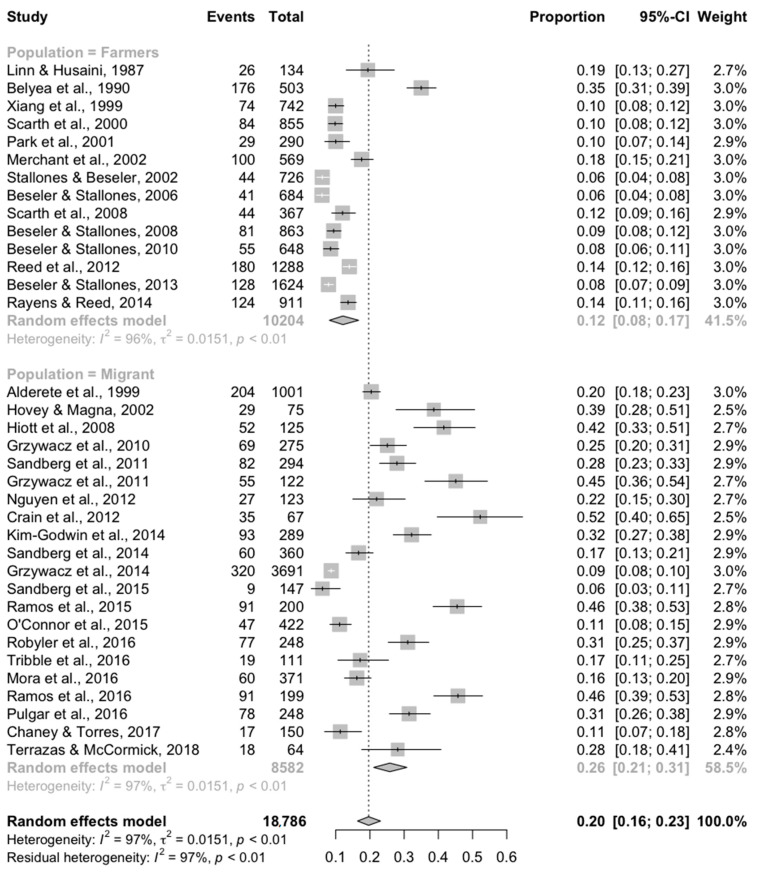
Results of the subgroup meta-analysis of the 35 studies conducted in the United States reporting a binary outcome (yes/no) using the CES-D/DR scale to measure depression in farming populations (“Farmers” = non-migrant farming population; “Migrant” = study restricted to migrant farmworkers).

Subgroup analysis by study population was separately conducted for studies reporting binary outcomes and those reporting continuous outcomes. For binary outcomes, results of the subgroup meta-analysis are reported in Figure 3 and Figure 4. Both subgroups contained substantial heterogeneity, indicating that a large amount of between-study variation remained after accounting for the study population. However, the confidence intervals of the point estimates did not overlap, indicating that although a large amount of between study variation remains after the sub-group analysis, the two study populations appear to have genuine differences in the prevalence of depression. For studies evaluating a non-migrant farming population (n = 14), the overall point estimate for the prevalence of depression was 12% (95% CI = 8–17%; *I*^2^ = 96%). For studies sampling from a migrant farmworker population (n = 21), the overall point estimate for the prevalence of depression was 26% (95% CI = 21–31%; *I*^2^ = 97%).

**Figure 5:**
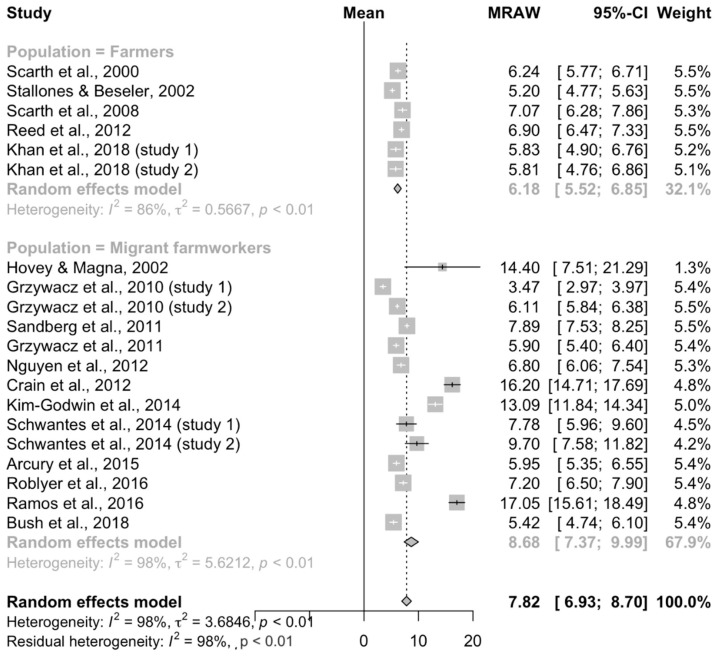
Results of the subgroup meta-analysis of the 20 studies conducted in the United States reporting a continuous outcome (raw mean score, MRAW) using the CES-D/DR scale to measure depression in farming populations.

Similarly, the subgroup analysis for studies reporting a continuous outcome (Figure 5) did not reduce the residual heterogeneity but did reveal point estimates between the population types that did not overlap. For studies evaluating a non-migrant farming population (n = 6), the mean scale score was 6.18 (95% CI = 5.52–6.85; *I*^2^ = 86%). For studies of migrant farmworkers (n = 14), the mean scale score was 8.68 (95% CI = 7.37–9.99; *I*^2^ = 98%).

## 4. Discussion

### 4.1. Summary of Results

A substantial body of evidence was found that aimed to estimate the prevalence of depression among farming populations. However, there were 14 studies from which data were not extracted based on poor reporting of the outcome, either failing to report a variance measure with continuous data, or not reporting the total study population. Full reporting of data is necessary to augment the value of primary research by allowing for inclusion in formal syntheses. Publishing all results in supplemental material online may aid in full reporting of study results, as well as adherence to reporting guidelines.

Numerous different self-reporting scales were used to evaluate depression within the included studies. While the majority of studies utilized the CES-D/DR scale, this was limited primarily to use in studies in the US. One reason for this may be that, within the US, the CES-D was translated into the Spanish language for use in migrant farmworker populations, which represented 62% of the included studies. Other scales were not used consistently, and therefore subgroup analysis by scale type was not possible. While consideration of standardized outcome reporting may be warranted in order to synthesize results across studies [20], it is also possible that these tools may be more or less appropriate for a given population [21]. However, the CES-D/DR was developed in English but has been modified for other languages [22]. There would be tremendous value in developing core outcome sets, including which scales would be suitable for these populations in this field of research with stakeholders (e.g., farmers, researchers, psychiatrists, and general practitioners) [20].

Overall, prevalence of depression appeared higher in the studies examining the migrant farmworker subgroup as compared to the non-migrant farming population group. Similarly, the mean CES-D/DR scale score appeared higher for studies in migrant farmworker populations. However, caution should be exercised in the interpretation of the exact point estimates in the analyses due to the substantial heterogeneity, indicating that much of the between-study variance remained unexplained. Heterogeneity may result from both contextual and methodological factors [23]. We suspect some important contextual factors may include gender, as it varied within the study populations; the majority of studies were conducted with study populations with males and females, with varying gender proportions. This has been shown in previous research; one scoping review reported that depression may be experienced differently based on gender, including farming populations [24]. Other contextual factors may include changes in disease trends over time, including willingness to report depression and depression symptomology. Recent attention to the importance of mental health may mean that respondents are more likely to participate and report symptomology in studies of depression. Interestingly, in our meta-analyses, only three studies (21%) in the non-migrant farming population were published after 2010, whereas 17 studies (81%) among the migrant farmworker population were published in the same timeframe, suggesting higher emphasis on migrant farmworkers.

There could be other contextual factors, such as the specific geographic location, farm type, and political changes, which have not been fully explored in the context of depression. Methodological differences between studies that can contribute to heterogeneity include selection bias, confounding bias, information bias, and variability in study execution.

Some studies have focused on adapting existing validated depression scales to account for contextual flux, adapting existing scales to reflect the context of farming. This has been done primarily in migrant farming populations, where language and circumstance may be barriers to participating in a survey assessing depression. Within non-migrant farming populations, it may be worthwhile to see whether revising an existing validated depression screening tool could allow for farming context to be captured, resulting in even more accurate depression estimates among non-migrant farmers. This may help to increase the response rates in randomly selected populations. Furthermore, using this type of validated tool broadly across regions and nations would allow for comparable data, and contribute to a more comprehensive understanding of the extent to which depression affects farmers worldwide.

### 4.2. Limitations of the Body of Evidence

Selection bias may contribute to increased heterogeneity within the meta-analysis, and also can impact the validity of the overall point estimate. In this analysis, half of the included studies used convenience sampling. Further, as half of the included studies were sampled using a convenience approach, approximately 55% did not report a response rate. This may lead to an underestimate of the prevalence of depression in these samples. Participants that respond to a convenience-based survey may be fundamentally different than those who are randomly sampled. Those who are depressed may be less likely to participate in a survey, compared to individuals without depression [25]. Within our study, half of the included studies used a convenience sampling approach, which is susceptible to selection bias and may account for some of this variability. In future studies, using a sampling frame constructed from members lists of commodity organizations, or governmental registries, could decrease the variability and strengthen the validity of the results.

Half of the included studies did not report the number of participants who experienced depression, but rather reported a percentage. As a result, we used the reported number of sampled participants as the denominator; if there were participants that did not complete the CES questions, this would result in an inflated denominator, and thus, an underestimated prevalence of depression. Including all participant data within written reports would ensure method transparency and allow for easier assessment and evaluations of studies.

### 4.3. Limitations of the Review

This review limited selection to articles published in English. It is likely that there is more research that has been conducted outside of English-speaking regions that was not captured.

## 5. Conclusions

There is a large body of evidence examining depression among farming populations. Despite the volume of research, there are challenges in determining an accurate point estimate. This is likely influenced by failure to report key study design and execution elements. Potential for confounding and selection biases are high with important differences in study populations, such as gender, commodity, and geographic region. While there are many small-scale studies examining depression among farming populations, there are a multitude of scales used, and they vary vastly in sample size. Future work would benefit from focusing on sampling methods that may produce results that are more representative of the broader farming communities. For example, using epidemiologic approaches to study design, and investing time and project funds to conducting studies using a random sampling approach could help to eliminate some of the heterogeneity within the data, and allow for better cross-study comparisons.

Lastly, not all published studies are thoroughly reported, and would benefit from following reporting guidelines. Ensuring that all published studies use the reporting guidelines is essential in order to conduct and interpret a useful meta-analysis. Not using reporting guidelines can leave significant gaps in our understanding, and contributes to the heterogeneity within the meta-analysis presented in this study.

Despite the heterogeneity within the meta-analyses, it is apparent that migrant farmworkers in the US experienced depression at an increased prevalence compared to farmers in the studies sampled. Furthermore, this study shows that, outside of the US, there is opportunity to increase the knowledge around the prevalence of depression among all farming populations. Through well-designed, executed, and reported epidemiologic studies, we can continue to improve our understanding of depression among farming populations worldwide.

## Figures and Tables

**Table 1:** Study characteristics for the 42 studies conducted in the United States using the CES-D/DR scale, included in the meta-analyses.

Study	Year of Conduct *	Study Population	Gender Composition (Proportion Female)	Outcome Metric **
Linn and Husaini, 1987 ***	1977–1979	Farmers	Mixed (58)	B
Belyea et al., 1990	1987	Farmers	Mixed (2)	B
Alderete et al., 1999	NR	Migrant farmworkers	Mixed (50)	B
Xiang et al., 1999	1993–1996	Farmers	Mixed (40)	B
Scarth et al., 2000	1993	Farmers	Men	B
Park et al., 2001	1995	Farmers	Men	B
Hovey and Magna, 2002	NR	Migrant farmworkers	Mixed (51)	B, C
Merchant et al., 2002	1994–1998	Farmers	Mixed (49)	B
Stallones and Beseler, 2002	1992–1997	Farmers	Mixed (40)	B, C
Beseler and Stallones, 2006	1992–1997	Farmers	Mixed (35)	B
Beseler and Stallones, 2008	NR	Farmers	Mixed (47)	B
Hiott et al., 2008	2003	Migrant farmworkers	Men	B
Scarth et al., 2008	1994	Farmers	Mixed (1)	B, C
Beseler and Stallones, 2010	1993–1995	Farmers	Mixed (49)	B
Grzywacz et al., 2010 (study 1)	2007	Migrant farmworkers	Mixed (9)	B, C
Grzywacz et al., 2010 (study 2)	2007	Migrant farmworkers	Mixed (22)	C
Grzywacz et al., 2011	2008	Migrant farmworkers	Mixed (11)	B, C
Sandberg et al., 2011	2009	Migrant farmworkers	Mixed (5)	B, C
Crain et al., 2012	2009	Migrant farmworkers	Men	B, C
Nguyen et al., 2012	2008	Migrant farmworkers	Mixed (10)	B, C
Reed et al., 2012	2002–2003	Farmers	Mixed (49)	B, C
Beseler and Stallones, 2013	1993–1997	Farmers	Mixed (47)	B
Grzywacz et al., 2014	2009–2010	Migrant farmworkers	Mixed (17)	B
Kim-Godwin et al., 2014	2007	Migrant farmworkers	Mixed (47)	B, C
Rayens and Reed, 2014	NR	Farmers	Mixed (50)	B
Sandberg et al., 2014	2010	Migrant farmworkers	Men	B
Schwantes et al., 2014 (Study 1)	NR	Migrant farmworkers	Men	C
Schwantes et al., 2014 (Study 2)	NR	Migrant farmworkers	Men	C
Arcury et al., 2015	2011–2013	Migrant farmworkers	Women	C
O’Connor et al., 2015	2005–2007	Migrant farmworkers	Men	B
Ramos et al., 2015	2013	Migrant farmworkers	Mixed (7)	B
Sandberg et al., 2015	2012	Migrant farmworkers	Men	B
Mora et al., 2016	2010	Migrant farmworkers	Men	B
Pulgar et al., 2016	2011–2012	Migrant farmworkers	Women	B
Ramos et al., 2016	2013	Migrant farmworkers	Mixed (7)	B, C
Robyler et al., 2016	2011–2012	Migrant farmworkers	Women	B, C
Tribble et al., 2016	2012–2014	Migrant farmworkers	Men	B
Chaney and Torres, 2017	2014	Migrant farmworkers	Mixed (55)	B
Bush et al., 2018	2013–2014	Migrant farmworkers	Mixed (NR)	C
Khan et al., 2018 (Study 1)	2015	Farmers	Men	C
Khan et al., 2018 (Study 2)	2015	Farmers	Men	C
Terrazas and McCormick, 2018	NR	Migrant farmworkers	Mixed (14)	B

* NR = not reported; ** B = binary outcome metric; C = continuous outcome metric; B, C = both metrics reported. *** See Supplementary Files for full reference list (File S1. Full Reference List).

**Table 2:** Results of the risk of bias assessment for the 42 included studies using a modified Quality Assessment of Diagnostic Accuracy Studies (QUADAS) tool.

QUADAS Item	N (%)
Target population explicitly stated	41/42 (97.6)
Sampling method:	
Random sampling	21/42 (50.0)
Convenience sampling	21/42 (50.0)
Described non-responders	4/42 (9.5)
Response rate:	
Not reported	23/42 (54.8)
Response rate: <70%	14/42 (33.3)
Response rate: 70–90%	5/42 (11.9)
Response rate: >90%	0/42 (0.0)
Description of age and gender of participants	40/42 (95.2)
Description of study population	41/42 (97.6)
Same mode of data collection for all participants	41/42 (97.6)
Reported the final sample size for their study population	41/42 (97.6)
Reported a denominator for the number of participants classified as having depression	21/42 (50.0)

## Supplementary Materials

The following are available online at https://www.mdpi.com/1660-4601/17/24/9376/s1, Table S1: Risk of Bias Form. Material S1: Full-text Exclusions. File S1: Full Reference List.
