# Supplementary material for: A Systematic Review and Meta-Analysis of Depression among Farming Populations Worldwide
Source: Int J Environ Res Public Health. 2020 Dec 15;17(24):9376. doi: 10.3390/ijerph17249376 (PMC7765209; doi:10.3390/ijerph17249376)
Supplement: Supplementary file 1 [file ijerph-17-09376-s001.pdf]

RefID: 1, There and Back Again: A Review of Residency and Return Migrations in Sharks, with Implications for Population Structure and Management. Chapman DD, Feldheim KA, Papastamatiou Y, Hueter RE

The overexploitation of sharks has become a global environmental issue in need of a comprehensive and multifaceted management response. Tracking studies are beginning to elucidate how shark movements shape the internal dynamics and structure of populations, which determine the most appropriate scale of these management efforts.

Tracked sharks frequently either remain in a restricted geographic area for an extended period of time (residency) or return to a previously resided-in area after making long-distance movements (site fidelity). Genetic studies have shown that some individuals of certain species preferentially return to their exact birthplaces (natal philopatry) or birth regions (regional philopatry) for either parturition or mating, even though they make long-distance movements that would allow them to breed elsewhere. More than 80 peer-reviewed articles, constituting the majority of published shark tracking and population genetic studies, provide evidence of at least one of these behaviors in a combined 31 shark species from six of the eight extant orders.

Residency, site fidelity, and philopatry can alone or in combination structure many coastal shark populations on finer geographic scales than expected based on their potential for dispersal. This information should therefore be used to scale and inform assessment, management, and conservation activities intended to restore depleted shark populations. Expected final online publication date for the Annual Review of Marine Science Volume 7 is January 03, 2015.

1. Was the target population explicitly stated or did you have to assume what it was?

   - Stated target population
   - had to assume target population

2. Which of the following is true regarding the sample of the study

   - ☐ entire target population
   - ☐ randomly selected sample
   - ☐ convenience sample
   - ☐ sample stated as representative of target population
   - ☐ not reported

3. Which of the following applies with regards to responders and nonresponders

   - ☐ reasons for non responders described
   - ☐ described non responders
   - ☐ compared responders and non responders
   - ☐ compared sample and target population
   - ☐ not reported

4. What was the response rate?

   - ≥ 90%
   - 70-90%
   - ≤ 70%
   - not reported

5. Was data primary from a prevalence study or pulled from a survey designed for another purpose?

   - prevalence study
   - another design
   - not reported

6. Was the same mode of data collection used for all participants?

   - Yes
   - No

Copyright © 2008-2020, Evidence Partners Inc., All Rights Reserved || Release Notes (https://www.manula.com/manuals/evidence-partners/release-notes/1/en/topic/distillersr)
Loading time: 0.327 seconds

7. Were the data collected directly using a validated scale?

- Yes
- No validated scale was used
- data collected from proxies or retrospectively from medical records
- not reported

8. Was there a description of the study population and setting that the participants were sampled from?

- Yes
- No

9. Was there a description of the gender and age of participants?

- Yes
- No

10. Did they report the final sample size or the number of participants that were analyzed?

- Yes
- No

11. report number of participants classified as having depression as a number rather than a percentage of their sample.

- Yes
- No

Full text not available

RefID: 142, Mental health self-care strategies of Mexican seasonal farm workers in Canada: an Ethnographic Study
A. Escrig
Level: 2, State: Excluded

RefID: 741, The impact of a natural disaster on physical and mental health: Offspring morbidity and crop loss as mediators of paternal testosterone, cortisol, and depression among Bolivian forager-farmers
B. C. Trumble, J. Stieglitz, A. Jaeggi, B. Beheim, M. Schwartz, H. Kaplan, M. Gurven
Level: 2, State: Excluded

RefID: 825, Informal Caregivers' Subjective Well-Being After a Training and Recreation Week: Multilevel Models for Longitudinal Data
C. Hetzel, M. Opfermann-Kersten, M. Holzer
Level: 2, State: Excluded

RefID: 911, Maharashtra government launches mental health programme to reduce suicide in farmers
C. Travasso
Level: 2, State: Excluded

RefID: 1402, Metabolic syndrome and mental health: A study of Australian farm men and women
N. Jeffery-Dawes, D. Mellor, S. Brumby
Level: 2, State: Excluded

RefID: 1412, A Predictive Model of Depression in Rural Elders-Decision Tree Analysis
S. E. Kim, S. A. Kim
Level: 2, State: Excluded

RefID: 1572, The Farming Family Work Environment: Consideration of a Hypothesised Model of Role Interference
C. McShane, F. Quirk, A. Swinbourne
Level: 2, State: Excluded

RefID: 2268, Mental health first aid training for Advisory and Extension Agents working with farmers
D. Gorman, D. Hossain, R. Eley, J. Couts
Level: 2, State: Excluded

RefID: 2313, Mental health and work in a national survey of farm managers in the United States
T. Alterman, J. Li, A. Steege, M. Petersen, C. Muntaner
Level: 2, State: Excluded

RefID: 2325, Depressive symptoms and contraceptive use among Latinas in an agricultural community
T. L. Barcellos, M. T. Stoecklin-Marois, T. E. Hennessy-Burt, M. B. Schenker
Level: 2, State: Excluded

RefID: 2665, Affective disorders among the rural population
V. N. S. de Snyder, M. D. Diaz-Perez
Level: 2, State: Excluded

RefID: 2959, Farm stress: A community development approach to mental health service delivery
N. Gerrard
Level: 2, State: Excluded

RefID: 2962, Mental health risk among rural adolescents
F. Bujak
Level: 2, State: Excluded

RefID: 3110, Farm Family Mental-Health Issues
J. L. Ellis, P. R. Gordon
Level: 2, State: Excluded

RefID: 3159, Mental-Health and Unemployment - Psychopathologic Approximation to Agricultural Lock-Outs - Spanish - Sevadiaz,A
L. Meyer
Level: 2, State: Excluded

RefID: 3188, Agriculture, Anxiety of the Whole World
R. Veylon
Level: 2, State: Excluded

RefID: 3282, A comparison of barriers to mental health support-seeking among farming and non-farming adults in rural South Australia
Melissa J. Hull, James Dollman, Kari Vallury, Kate M. Fennell, Martin Jones
Level: 2, State: Excluded

RefID: 3878, Sowing the seeds of hope : responding to the mental health needs of farm families : rural services directory

Level: 2, State: Excluded

RefID: 3890, Responding to farm stress produced by Tommy Haggas & Ryan Powell ; a Loras College Production

Level: 2, State: Excluded

RefID: 3908, Optimism and well-being--in rural communities
C. Day
Level: 2, State: Excluded

RefID: 3915, Psychological distress and size of place: the epidemiology of rural economic stress
D. R. Hoyt, D. O'Donnell, K. Y. Mack
Level: 2, State: Excluded

RefID: 3935, Inpatient mental health services in rural areas: an interregional comparison
M. O. Wagenfeld, H. F. Goldsmith, D. Stiles, R. W. Manderscheid
Level: 2, State: Excluded

RefID: 3954, Consumption patterns, hardship, and stress among farm households
L. M. Lobao, K. Meyer
Level: 2, State: Excluded

RefID: 3967, Delivery of mental health services is a special problem in rural areas
R. L. Pitzer
Level: 2, State: Excluded

RefID: 3976, The well-being of migrant farm workers in New York State
P. S. K. Chi
Level: 2, State: Excluded

RefID: 3980, Farm families and stress: one year later
J. Obst
Level: 2, State: Excluded

RefID: 3984, Farmers in distress: how veterinarians can help out
R. T. Williams
Level: 2, State: Excluded

RefID: 4030, Mental health response to the farm foreclosure crisis
D. S. Hargrove
Level: 2, State: Excluded

RefID: 4033, Stress on the farm. 3. Understanding depression--yours and theirs
R. Weigel, S. Mays, B. Abbott
Level: 2, State: Excluded

RefID: 4066, The contribution of agricultural communities to mental health
H. L. Waring
Level: 2, State: Excluded

RefID: 4081, Depression in rural America
Linda Siderius, Sherry Leach

Level: 2, State: Excluded

RefID: 4110, Mental health in rural and urban environments
S. D. Webb
Level: 2, State: Excluded

RefID: 4118, Depressed area chooses 'COP' over 'cop out'
A. S. Holmes, B. Sorter
Level: 2, State: Excluded

RefID: 4159, Depression : cause, effect, remedy : Chapter III. Agriculture
Meno Kammerhoff
Level: 2, State: Excluded

RefID: 4162, What is the chief cause of the farm depression? : publication extracts which present diversified viewpoints on the question

Level: 2, State: Excluded

RefID: 4164, What is the chief cause of the farm depression?

Level: 2, State: Excluded

RefID: 4189, The incidence of farming prosperity and depression; a survey of conditions in England to-day, [by] D. Skilbeck and M. Messer
D. Skilbeck, M. Messer
Level: 2, State: Excluded

RefID: 6556, Major depression in a nonclinical sample. Demographic and clinical risk factors for first onset
W. Coryell, J. Endicott, M. Keller
Level: 2, State: Excluded

RefID: 6946, [Not Available]
A. Soldi, M. Alberti Claudi
Level: 2, State: Excluded

RefID: 6947, [Not Available]
V. Lund, C. Faurholt
Level: 2, State: Excluded

RefID: 6948, [Not Available]
A. Albarracin Teulon
Level: 2, State: Excluded

RefID: 7854, Understanding depression: yours and your family's

B. H. Frazier
Level: 2, State: Excluded

RefID: 7871, Let go of your depression
R. J. Fetsch
Level: 2, State: Excluded

RefID: 7941, The major causes of the depression. By Gardiner C. Means

Level: 2, State: Excluded

Not available in English

RefID: 402, Some aspects of circular depressive disorders pathomorphosis at present
N. G. Pshuk, I. S. Pototska, O. O. Belov
Level: 2, State: Excluded

RefID: 517, Lifestyle, Mental Health and Gender Conditions in Rural Contexts: A Study within Agraria Reform Settlements of Northeastern Brazil
J. F. Leite, M. Dimenstein, C. B. Dantas, E. L. Silva, J. P. S. Macedo, A. P. de Sousa
Level: 2, State: Excluded

RefID: 603, Evaluation of psycho-organisational constraints according to Karasek's Model among workers of 14 private sector companies in Tunisia
I. Magroun, H. Ghannouchi, S. Fehri, M. Chatti, F. Ben Salah, H. Nouaigui
Level: 2, State: Excluded

RefID: 1102, Construct and validation of a quality of life's scale for older French people
S. Petit, V. Bergua, K. Peres, J. Bouisson, M. Koleck
Level: 2, State: Excluded

RefID: 1323, The investigation of risk factors which predict postpartum depression in seasonal farm workers
M. Nebioglu, S. Akbaba, F. Kabalcioglu, Y. Eroglu, M. Gultekin
Level: 2, State: Excluded

RefID: 1842, Changes in perceived health in war-displaced population, Ayacucho, Peru: 1980-2004
J. M. Medina, S. Lopez-Moreno
Level: 2, State: Excluded

RefID: 1843, People's experiences in a natural environment in the Vredefort Dome, South Africa: Implications for spatial development
V. Roos, H. Coetzee, K. Puren
Level: 2, State: Excluded

RefID: 1991, IRREGULAR MIGRATION AND SOCIAL ISOLATION Tamaulipas' Undocumented Farm Workers in the United States
S. P. I. Palacios
Level: 2, State: Excluded

RefID: 2084, Mental health and wellbeing in Icelandic farmers
K. Tomasson, G. Guomundsson
Level: 2, State: Excluded

RefID: 2185, Trend and factors influencing suicides in rural areas of Kermanshah (in Iran) during 7 years (2000-2006)
P. Safaie, K. Jahangiri, N. Barkhordar, N. Barkhordar
Level: 2, State: Excluded

RefID: 2363, Working and health conditions and preventive measures in a random sample of 5000 workers in the Veneto Region examined by telephone interview
G. Mastrangelo, S. Perticaroli, G. Campo, G. Priolo, A. Leva, D. de Merich, G. Marangi, U. Fedeli, E. Fadda, L. Scoizzato, L. Marchiori
Level: 2, State: Excluded

RefID: 2680, A cross-sectional study about mental health of farm-workers
N. M. X. Faria, L. A. Facchini, A. G. Fassa, E. Tomasi
Level: 2, State: Excluded

RefID: 6195, [A cross-sectional study about mental health of farm-workers from Serra Gaucha (Brazil)]
N. M. Faria, L. A. Facchini, A. G. Fassa, E. Tomasi
Level: 2, State: Excluded

Not a primary study

RefID: 65, Suicide An Unrecognized Epidemic Among Farmers
M. S. Jones, D. B. Reed, M. L. Hunt
Level: 2, State: Excluded

RefID: 71, Inequity amplified: climate change, the Australian farmer, and mental health
M. Shorthouse, L. Stone
Level: 2, State: Excluded

RefID: 121, Agriculture, Anxiety, and Amazonification: Creative Adaptation and Resistance in Risky Rural and Urban Landscapes
B. Janssen, M. Styles
Level: 2, State: Excluded

RefID: 133, Longitudinal study of health, disease and access to care in rural Victoria: the Crossroads-II study: methods
K. M. Glenister, L. Bourke, L. Bolitho, S. Wright, S. Roberts, W. Kemp, L. Rhode, R. Bhat, S. Tremper, D. J. Magliano, M. Morgan, R. Marino, W. Adam, D. Simmons
Level: 2, State: Excluded

RefID: 160, 'Farming on the Edge': Wellbeing and Participation in Agri-Environmental Schemes
H. Saxby, M. Gkartzios, K. Scott
Level: 2, State: Excluded

RefID: 227, Mothers, wives, and farmers: Stories of women 'gone mad'
K. J. Millondaga
Level: 2, State: Excluded

RefID: 247, Migrant Workers and Their Occupational Health and Safety
S. C. Moyce, M. Schenker
Level: 2, State: Excluded

RefID: 257, The Care Economy in Post-Reform China: Feminist Research on Unpaid and Paid Work and Well-Being
R. Connelly, X. Y. Dong, J. Jacobsen, Y. H. Zhao
Level: 2, State: Excluded

RefID: 340, Pesticides: A Case Domain for Environmental Neuroethics
L. Y. Cabrera
Level: 2, State: Excluded

RefID: 374, Farmers' suicide and agrarian crisis: Social policy and public mental health
A. Das
Level: 2, State: Excluded

RefID: 535, Prevention of farmer suicides: Greater need for state role than for a mental health professional's role
T. S. S. Rao, M. R. Gowda, K. Ramachandran, C. Andrade
Level: 2, State: Excluded

RefID: 543, A Gendered Model of the Peasant Household: Time Poverty and Farm Production in Rural Mozambique
D. Arora, C. Rada
Level: 2, State: Excluded

RefID: 546, Comparison of the Center for Epidemiology Studies Depression Scale and Beck Depression Inventory for Research with Latinas
M. Bogardus
Level: 2, State: Excluded

RefID: 562, Participation in Social Activities and the Association with Socio-Demographic and Health-Related Factors among Community-Dwelling Older Adults in Jamaica
D. Willie-Tyndale, D. Holder-Nevins, K. Mitchell-Fearon, K. James, H. Laws, N. K. Waldron, D. Eldemire-Shearer
Level: 2, State: Excluded

RefID: 697, Social, Occupational, and Spatial Exposures and Mental Health Disparities of Working-Class Latinas in the US
Y. C. Hsieh, Y. Apostolopoulos, K. Hatzudis, S. Sonmez
Level: 2, State: Excluded

RefID: 894, "A Different Kettle of Fish": Mental health strategies for Australian fishers, and farmers
T. King, S. Kilpatrick, K. Willis, C. Speldewinde
Level: 2, State: Excluded

RefID: 974, Suicide in India
S. Aggarwal
Level: 2, State: Excluded

RefID: 1240, Are the Predictors of Work Absence Following a Work-Related Injury Similar for Musculoskeletal and Mental Health Claims?
P. M. Smith, O. Black, T. Keegel, A. Collie
Level: 2, State: Excluded

RefID: 1441, Should Mental Health Interventions Be Locally Grown or Factory-Farmed?
G. E. Simon, E. J. Ludman
Level: 2, State: Excluded

RefID: 1759, Improving the mental health of rural New South Wales communities facing drought and other adversities
C. R. Hart, H. L. Berry, A. M. Tonna
Level: 2, State: Excluded

RefID: 1801, Does intergenerational mobility shape psychological distress? Sorokin revisited
J. N. Houle, M. A. Martin
Level: 2, State: Excluded

RefID: 1847, Climate Change and Farmers' Mental Health: Risks and Responses
H. L. Berry, A. Hogan, J. Owen, D. Rickwood, L. Fragar
Level: 2, State: Excluded

RefID: 1898, Farmers' Suicide in India: Implications for Public Mental Health
A. Das
Level: 2, State: Excluded

RefID: 2136, Country Living, Country Dying: Rural Suicides in New Zealand, 1900-1950
J. C. Weaver, D. Munro
Level: 2, State: Excluded

RefID: 2697, Depressed farmers should have access to firearms restricted

Level: 2, State: Excluded

RefID: 3003, The Mexican-American Migrant Farmworker Family - Mental-Health Issues
M. L. D. Siantz
Level: 2, State: Excluded

RefID: 3184, Agriculture and the Depressed Areas
M. Olson
Level: 2, State: Excluded

RefID: 3701, Health in rural Canada / edited by Judith C. Kulig and Allison M. Williams
Judith Celene Kulig, Allison Williams
Level: 2, State: Excluded

RefID: 3790, Depression and Poverty Among Rural Women: A Relationship of Social Causation or Social Selection
Leigh A. Simmons, Bonnie Braun, Richard Charnigo, Jennifer R. Havens, David W. Wright
Level: 2, State: Excluded

RefID: 3856, Critical issues in rural health / edited by Nina Glasgow, Lois Wright Morton, Nan E. Johnson
Nina Glasgow, Nan E. Johnson, Lois Wright Morton
Level: 2, State: Excluded

RefID: 3858, Economic hardship, religion and mental health during the midwestern farm crisis
Katherine Meyer, Linda Lobao
Level: 2, State: Excluded

RefID: 3956, Financial strain and depression among farm operators: the role of perceived economic hardship and personal control
P. S. Armstrong, M. D. Schulman
Level: 2, State: Excluded

RefID: 5001, Reducing stress to minimize injury: the nation's first employee assistance program for dairy farmers
S. Dickens, E. Dotter, M. Handy, L. Waterman
Level: 2, State: Excluded

RefID: 5139, Active coping, personal satisfaction, and attachment to land in older African-American farmers

S. A. Maciuba, S. C. Westneat, D. B. Reed
Level: 2, State: Excluded

RefID: 5648, The interrelation between organophosphate toxicity and the epidemiology of depression and suicide
K. Jaga, C. Dharmani
Level: 2, State: Excluded

RefID: 5689, Depression and pesticide exposures in female spouses of licensed pesticide applicators in the agricultural health study cohort
C. Beseler, L. Stallones, J. A. Hoppin, M. C. Alavanja, A. Blair, T. Keefe, F. Kamel
Level: 2, State: Excluded

RefID: 6117, Forensic psychiatric services in British Columbia
D. Eaves, D. Lamb, G. Tien
Level: 2, State: Excluded

RefID: 6217, The occupational health status of hired farm workers
D. Villarejo, S. L. Baron
Level: 2, State: Excluded

RefID: 6581, Mental health and illness nursing in rural Vermont: case illustration of a farm family
B. P. Hamel-Bissell
Level: 2, State: Excluded

RefID: 6599, Psychology and rural America. Current status and future directions
J. D. Murray, P. A. Keller
Level: 2, State: Excluded

RefID: 6801, Urbanicity and depression reconsidered. The evidence regarding depressive symptomatology
J. A. Neff
Level: 2, State: Excluded

RefID: 7598, High risk of depression among low-income women raises awareness about treatment options
Amy Block Joy, Mark Hudes
Level: 2, State: Excluded

Does not attempt to quantify depression in a farming population

RefID: 74, Water torture: Unravelling the psychological distress of irrigators in Australia
S. A. Wheeler, A. Zuo, A. Loch
Level: 2, State: Excluded

RefID: 78, Burnout and hopelessness among farmers: The Farmers Stressors Inventory
D. Truchot, M. Andela
Level: 2, State: Excluded

RefID: 84, Mental Health Conceptualization and Resilience Factors in the Kalasha Youth: An Indigenous Ethnic and Religious Minority Community in Pakistan
F. R. Choudhry, T. M. Khan, M. S. A. Park, K. J. Golden
Level: 2, State: Excluded

RefID: 85, Online assessment of suicide stigma, literacy and effect in Australia's rural farming community
A. J. Kennedy, S. A. Brumby, V. L. Versace, T. Brumby-Rendell
Level: 2, State: Excluded

RefID: 97, Internal migration and mental health of the second generation. The case of Turin in the age of the Italian economic miracle
M. Cardano, C. Scarinzi, G. Costa, A. d'Errico
Level: 2, State: Excluded

RefID: 116, "The Masks We Wear": A Qualitative Study of Suicide in Australian Farmers
L. Kunde, K. Kolves, B. Kelly, P. Reddy, D. de Leo
Level: 2, State: Excluded

RefID: 124, Overview on the Burnout Rate of Romanian Farmers
I. Zaharia, L. Reissig, G. Fintineru, A. M. Iorga
Level: 2, State: Excluded

RefID: 130, The effects of climate variability on psychological well-being in India
S. Pailler, M. Tsaneva
Level: 2, State: Excluded

RefID: 138, Integration anxiety: The cognitive isolation of climate change
K. M. Findlater, S. D. Donner, T. Satterfield, M. Kandlikar
Level: 2, State: Excluded

RefID: 141, Evolving parent-adult child relations: location of multiple children and psychological well-being of older adults in China
Z. Lin, F. Chen
Level: 2, State: Excluded

RefID: 149, Work routines moderate the association between eveningness and poor psychological well-being

F. G. Carvalho, C. M. de Souza, M. P. L. Hidalgo
Level: 2, State: Excluded

RefID: 151, What's up with the self-employed? A cross-national perspective on the self-employed's work-related mental well-being
J. Gevaert, D. De Moortel, M. Wilkens, C. Vanroelen
Level: 2, State: Excluded

RefID: 171, Length of Stay of Psychiatric Admissions in a Tertiary Care Hospital
M. Basnet, N. Sapkota, S. Limbu, D. Baral
Level: 2, State: Excluded

RefID: 175, Case Control Study of Impulsivity, Aggression, Pesticide Exposure and Suicide Attempts Using Pesticides among Farmers
C. P. Lyu, J. R. Pei, L. C. Beseler, Y. L. Li, J. H. Li, M. Ren, L. Stallones, S. P. Ren
Level: 2, State: Excluded

RefID: 185, Farmers' perceptions of coexistence between agriculture and a large scale coal seam gas development
N. I. Huth, B. Cocks, N. Dalgliesh, P. L. Poulton, O. Marinoni, J. N. Garcia
Level: 2, State: Excluded

RefID: 204, The prevalence, age-of-onset and the correlates of DSM-IV psychiatric disorders in the Tianjin Mental Health Survey (TJMHS)
H. Yin, G. Xu, H. Tian, G. Yang, K. J. Wardenaar, R. A. Schoevers
Level: 2, State: Excluded

RefID: 211, Quality of life and mental health among Thai older workers in community enterprises
S. Hanklang, P. Ratanasiripong, S. Naksranoi, S. Sathira-Anant, K. Patanasri
Level: 2, State: Excluded

RefID: 215, Generating toxic landscapes: impact on well-being of cotton farmers in Telangana, India
N. K. Kannuri, S. Jadhav
Level: 2, State: Excluded

RefID: 216, Farmers's perception and strategies for the development of sustainable livelihoods in disaster prone areas
S. Anantanyu,Suwarto,Suminah
Level: 2, State: Excluded

RefID: 224, Psychological distance of climate change and mental health risks assessment of smallholder farmers in Northern Ghana: Is habituation a threat to climate change?
J. W. Acharibasam, S. W. Anuga
Level: 2, State: Excluded

RefID: 228, Emotions, attitudes, and appraisal in the management of climate-related risks by fish farmers in Northern Thailand
L. Lebel, P. Lebel
Level: 2, State: Excluded

RefID: 230, Toxic Effects of Exposure to Pesticides in Farm Workers in Navolato, Sinaloa (Mexico)
J. G. Galindo-Reyes, H. Alegria
Level: 2, State: Excluded

RefID: 234, Job Resources and Work Engagement among Finnish Dairy Farmers
M. K. Kallioniemi, J. Kaseva, C. L. Kolstrup, A. Simola, H. R. Kymalainen
Level: 2, State: Excluded

RefID: 238, Exploring perspectives of well-being in Latina/o migrant workers
R. B. Herbst, R. M. Gonzalez-Guarda
Level: 2, State: Excluded

RefID: 243, Association among Occupational Stress factors and Performance at workplace among Agricultural Research Sector Employees at Hyderabad, India
K. D. V. Prasad, R. Vaidya, V. A. Kumar
Level: 2, State: Excluded

RefID: 249, Migrant perinatal depression study: a prospective cohort study of perinatal depression on the Thai-Myanmar border
G. Fellmeth, E. H. Plugge, V. Carrara, M. Fazel, M. May, Y. Phichitphadungtham, M. Pimanpanarak, N. K. Wai, O. Mu, P. Charunwatthana, F. Nosten, R. Fitzpatrick, R. McGready
Level: 2, State: Excluded

RefID: 253, The salivary alpha amylase awakening response is related to over-commitment
P. Eddy, E. H. Wertheim, M. W. Hale, B. J. Wright
Level: 2, State: Excluded

RefID: 260, Health and Safety in Organic Farming: A Qualitative Study
F. S. Mas, A. J. Handal, R. E. Rohrer, E. T. Viteri
Level: 2, State: Excluded

RefID: 261, Oral history and farmworker studies
J. Hagood, C. Schriemer
Level: 2, State: Excluded

RefID: 269, Farm suicides in New Zealand, 2007-2015: A review of coroners' records
A. L. Beautrais
Level: 2, State: Excluded

RefID: 280, Criteria and indicators for foot and mouth disease control strategy decision-making in Asia-Oceania countries
E. Kim, T. Carpenter, S. Rowanowski, N. Cogger
Level: 2, State: Excluded

RefID: 303, Epidemiology of severe mental illness in Hunan province in central China during 2014-2015: A multistage cross-sectional study
D. X. Wang, J. Ma, L. H. Tan, Y. Chen, X. S. Li, X. F. Tian, X. H. Zhou, X. J. Liu
Level: 2, State: Excluded

RefID: 310, Depressive symptoms among land expropriated farmers: evidence from China
Y. Mizushima
Level: 2, State: Excluded

RefID: 319, Factors increasing the risk for psychosocial stress among Korean adults living in rural areas: using generalized estimating equations and mixed models
J. H. Nam, M. S. Lim, H. K. Choi, J. Y. Kim, S. K. Kim, S. S. Oh, S. B. Koh, H. T. Kang
Level: 2, State: Excluded

RefID: 350, "Do it All by Myself": A Salutogenic Approach of Masculine Health Practice Among Farming Men Coping With Stress
P. Roy, G. Tremblay, S. Robertson, J. Houle
Level: 2, State: Excluded

RefID: 381, Farmer suicides: a qualitative study from Australia
M. Perceval, K. Kolves, P. Reddy, D. De Leo
Level: 2, State: Excluded

RefID: 385, Belonging and Mental Wellbeing Among a Rural Indian-Canadian Diaspora: Navigating Tensions in "Finding a Space of Our Own"
C. S. Caxaj, N. K. Gill
Level: 2, State: Excluded

RefID: 415, Suicide attempters with high and low suicide intent: Different populations in rural China
B. P. Liu, X. T. Wang, C. X. Jia
Level: 2, State: Excluded

RefID: 441, Influence of socio-economic and psychosocial factors on food insecurity and nutritional status of older adults in FELDA settlement in Malaysia
R. S. Hudin, S. Shahar, N. Ibrahim, H. M. Yahaya
Level: 2, State: Excluded

RefID: 446, Oxidative stress and cholinesterase depression among farm workers occupationally exposed to ptsticides in India
M. Fareed, C. N. Kesavachandran, V. Bihari, R. Kamal, M. Kuddus

Level: 2, State: Excluded

RefID: 457, Comparison of neurological health outcomes between two adolescent cohorts exposed to pesticides in Egypt
A. A. Ismail, M. R. Bonner, O. Hendy, G. A. Rasoul, K. Wang, J. R. Olson, D. S. Rohlman
Level: 2, State: Excluded

RefID: 465, Climate change threats to family farmers' sense of place and mental wellbeing: A case study from the Western Australian Wheatbelt
N. R. Ellis, G. A. Albrecht
Level: 2, State: Excluded

RefID: 471, Immigration policies and mental health morbidity among Latinos: A state-level analysis
M. L. Hatzenbuehler, S. J. Prins, M. Flake, M. Philbin, M. S. Frazer, D. Hagen, J. Hirsch
Level: 2, State: Excluded

RefID: 510, GoodYarn: building mental health literacy in New Zealand's rural workforce
K. Morgaine, L. Thompson, K. Jahnke, R. Llewellyn
Level: 2, State: Excluded

RefID: 530, Prevalence of serious psychological distress among slaughterhouse workers at a United States beef packing plant
J. H. Leibler, P. A. Janulewicz, M. J. Perry
Level: 2, State: Excluded

RefID: 564, Communal farmers' perception of drought in South Africa: Policy implication for drought risk reduction
Y. T. Bahta, A. Jordaan, F. Muyambo
Level: 2, State: Excluded

RefID: 581, Depression among migrant workers in Al-Qassim, Saudi Arabia
W. Nadim, A. AlOtaibi, A. Al-Mohaimeed, M. Ewid, M. Sarhandi, J. Saquib, K. Alhumdi, A. Alharbi, A. Taskin, M. Migdad, J. Alshammari, S. Alharbi, N. Saquib
Level: 2, State: Excluded

RefID: 620, The Red Blood Cell Acetylcholinesterase Levels of Depressive Patients with Suicidal Behavior in an Agricultural Area
V. Altinyazar, F. B. Sirin, R. Sutcu, I. Eren, I. K. Omurlu
Level: 2, State: Excluded

RefID: 634, Exposure to pesticides and mental disorders in a rural population of Southern Brazil
E. Campos, V. D. P. da Silva, M. S. C. de Mello, U. B. Otero
Level: 2, State: Excluded

RefID: 659, Analysis of factors associated with hesitation to restart farming after depopulation of

animals due to 2010 foot-and-mouth disease epidemic in Japan
H. Kadowaki, T. Kayano, T. Tobinaga, A. Tsutsumi, M. Watari, K. Makita
Level: 2, State: Excluded

RefID: 661, "Watching the bank balance build up then blow away and the rain clouds do the same": A thematic analysis of South Australian farmers' sources of stress during drought
K. M. Fennell, C. E. Jarrett, L. J. Kettler, J. Dollman, D. A. Turnbull
Level: 2, State: Excluded

RefID: 672, Prevalence and treatment coverage for depression: a population-based survey in Vidarbha, India
R. Shidhaye, S. P. Gangale, V. Patel
Level: 2, State: Excluded

RefID: 719, A case-control study of psychological distress in survivors of farmers' suicides in Wardha District in central India
M. C. Bhise, P. B. Behere
Level: 2, State: Excluded

RefID: 772, Occupational differences in suicide mortality among Japanese men of working age
K. Wada, H. Eguchi, D. Prieto-Merino, D. R. Smith
Level: 2, State: Excluded

RefID: 807, Suicide Among Farmers in France: Occupational Factors and Recent Trends
C. Bossard, G. Santin, I. G. Canu
Level: 2, State: Excluded

RefID: 819, Geographic variation in suicide rates in Australian farmers: Why is the problem more frequent in Queensland than in New South Wales?
U. Arnautovska, S. McPhedran, B. Kelly, P. Reddy, D. De Leo
Level: 2, State: Excluded

RefID: 836, Prevalence of Diabetes and Health-Related Quality of Life Among Rural-to-Urban Nong Zhuan Fei Migrants in an Urban Area of Northern China, 2013
S. Yan, X. C. Hong, H. Q. Yu, Z. Yang, S. Y. Liu, W. Quan, J. K. Xu, L. Y. Zhu, W. L. Cheng, H. Xiao, H. Kitzman-Ulrich, M. J. DeHaven
Level: 2, State: Excluded

RefID: 848, Reflecting the interface between rural work and mental health of citriculture workers
A. C. Santos, S. A. Menta
Level: 2, State: Excluded

RefID: 851, Maternal depression and malnutrition in children in southwest Uganda: a case control study
S. Ashaba, G. Z. Rukundo, F. Beinempaka, M. Ntaro, J. C. LeBlanc
Level: 2, State: Excluded

RefID: 878, Functional Disability and Social Conflict Increase Risk of Depression in Older Adulthood Among Bolivian Forager-Farmers
J. Stieglitz, E. Schniter, C. von Rueden, H. Kaplan, M. Gurven
Level: 2, State: Excluded

RefID: 908, Housing and Neighborhood Characteristics and Latino Farmworker Family Well-Being
T. A. Arcury, G. Trejo, C. K. Suerken, J. G. Grzywacz, E. H. Ip, S. A. Quandt
Level: 2, State: Excluded

RefID: 934, Effect of subjective economic status on psychological distress among farmers and non-farmers of rural China
D. J. Feng, L. Q. Ji, L. Z. Xu
Level: 2, State: Excluded

RefID: 1014, Pesticide exposure and health conditions of terrestrial pesticide applicators in Cordoba Province, Argentina
M. Butinof, R. A. Fernandez, M. I. Stimolo, M. J. Lantieri, M. Blanco, A. L. Machado, G. Franchini, M. D. Diaz
Level: 2, State: Excluded

RefID: 1031, The Impact of Drought on Mental Health in Rural and Regional Australia
B. Edwards, M. Gray, B. Hunter
Level: 2, State: Excluded

RefID: 1103, Work Organization and Health Among Immigrant Women: Latina Manual Workers in North Carolina
T. A. Arcury, J. G. Grzywacz, H. Y. Chen, D. C. Mora, S. A. Quandt
Level: 2, State: Excluded

RefID: 1105, Association between pesticide exposure and suicide rates in Brazil
N. M. X. Faria, A. G. Fassa, R. D. Meucci
Level: 2, State: Excluded

RefID: 1117, Stress and Sociocultural Factors Related to Health Status Among US-Mexico Border Farmworkers
S. Carvajal, C. Kibor, D. McClelland, M. Ingram, J. G. de Zapien, E. Torres, F. Redondo, K. Rodriguez, R. Rubio-Goldsmith, J. Meister, C. Rosales
Level: 2, State: Excluded

RefID: 1125, The Prevalence and Correlates of Lifetime Psychiatric Disorders and Trauma Exposures in Urban and Rural Settings: Results from the National Comorbidity Survey Replication (NCS-R)
J. S. McCall-Hosenfeld, S. Mukherjee, E. B. Lehman
Level: 2, State: Excluded

RefID: 1131, Work Ability Index (WAI) and Its Health-related Determinants among Iranian Farmers Working in Small Farm Enterprises
A. Rostamabadi, A. Mazloumi, A. R. Foroushani
Level: 2, State: Excluded

RefID: 1152, Health Needs Assessment of Older People in an Agricultural Plantation
N. C. Din, S. E. Ghazali, N. Ibrahim, M. Ahmad, Z. Said, A. R. Ghazali, R. Razali, S. Shahar
Level: 2, State: Excluded

RefID: 1155, Suicide Mortality Among Agricultural Workers in a Region With Intensive Tobacco Farming and Use of Pesticides in Brazil
N. Krawczyk, A. Meyer, M. Fonseca, J. Lima
Level: 2, State: Excluded

RefID: 1169, The effectiveness of a community-based health promotion program for rural elders: A quasi-experimental design
J. Wang, C. Y. Chen, L. J. Lai, M. L. Chen, M. Y. Chen
Level: 2, State: Excluded

RefID: 1207, Drought as a mental health exposure
L. V. Obrien, H. L. Berry, C. Coleman, I. C. Hanigan
Level: 2, State: Excluded

RefID: 1224, A regional approach to understanding farmer suicide rates in Queensland
U. Arnautovska, S. McPhedran, D. De Leo
Level: 2, State: Excluded

RefID: 1261, Are social security policies for Chinese landless farmers really effective on health in the process of Chinese rapid urbanization? a study on the effect of social security policies for Chinese landless farmers on their health-related quality of life
Y. Liang, W. Y. Lu, W. Wu
Level: 2, State: Excluded

RefID: 1274, Association between pain and agricultural workload
L. P. Rocha, M. R. Cezar-Vaz, M. C. V. de Almeida, D. R. Piexak, C. A. Bonow
Level: 2, State: Excluded

RefID: 1300, Health symptoms related to pesticide exposure and agricultural tasks among rice farmers from northern Thailand
R. Sapbamrer, S. Nata
Level: 2, State: Excluded

RefID: 1328, Miseries Suffered, Unvoiced, Unknown? Communication of Suicidal Intent by Men in "Rural" Queensland, Australia
S. McPhedran, D. De Leo

Level: 2, State: Excluded

RefID: 1342, Characteristics of Chinese rural young suicides by pesticides
J. Zhang, Z. Y. Li
Level: 2, State: Excluded

RefID: 1415, Alcohol Consumption, Obesity, and Psychological Distress in Farming Communities-An Australian Study
S. Brumby, A. Kennedy, A. Chandrasekara
Level: 2, State: Excluded

RefID: 1425, Stress, Depression and Coping among Latino Migrant and Seasonal Farmworkers
S. B. Winkelman, E. H. Chaney, J. W. Bethel
Level: 2, State: Excluded

RefID: 1428, Prevalence and Risk Factor of Neck Pain in Elderly Korean Community Residents
K. M. Son, N. H. Cho, S. H. Lim, H. A. Kim
Level: 2, State: Excluded

RefID: 1438, Loneliness and depression among the elderly in an agricultural settlement: Mediating effects of social support
Wmywm Azam, N. C. Din, M. Ahmad, S. E. Ghazali, N. Ibrahim, Z. Said, A. R. Ghazali, S. Shahar, R. Razali, T. Maniam
Level: 2, State: Excluded

RefID: 1458, Battling Discrimination and Social Isolation: Psychological Distress Among Latino Day Laborers
N. J. Negi
Level: 2, State: Excluded

RefID: 1476, Factors associated with health-related quality of life among Indian women in mining and agriculture
M. S. D'Souza, S. N. Karkada, G. Somayaji
Level: 2, State: Excluded

RefID: 1549, Farmers' stress and coping in a time of drought
K. M. Gunn, L. J. Kettler, G. L. A. Skaczkowski, D. A. Turnbull
Level: 2, State: Excluded

RefID: 1578, Psychological Morbidity of Farmers and Non-farming Population: Results from a UK Survey
B. Hounsome, R. T. Edwards, N. Hounsome, G. Edwards-Jones
Level: 2, State: Excluded

RefID: 1589, Health and aging in elderly farmers: the AMI cohort
K. Peres, F. Matharan, M. Allard, H. Amieva, I. Baldi, P. Barberger-Gateau, V. Bergua, I.

Bourdel-Marchasson, C. Delcourt, A. Foubert-Samier, A. Fourrier-Reglat, M. Gaimard, S. Laberon, C. Maubaret, V. Postal, C. Chantal, M. Rainfray, N. Rascle, J. F. Dartigues
Level: 2, State: Excluded

RefID: 1594, Sickness absence due to mental health disorders-a societal perspective
C. A. M. Roelen, W. van Rhenen, P. C. Koopmans, U. Bultmann, J. W. Groothoff, J. J. L. van der Klink
Level: 2, State: Excluded

RefID: 1604, The psychosocial stress model for Thai contract farmers under globalization: a Path analysis model
C. Kaewanuchit, C. Muntaner, S. Dendoung, R. Labonte, C. Suttawet, W. Chiengkul
Level: 2, State: Excluded

RefID: 1683, Health Status of Migrant Farmworkers in the Shenandoah Valley
N. Kelly, D. Glick, P. Kulbok, L. Clayton, V. Rovnyak
Level: 2, State: Excluded

RefID: 1693, Family farming workers mental health in a microrregion in southern Brazil
A. R. Poletto, L. A. Gontijo
Level: 2, State: Excluded

RefID: 1760, Rapid change, climate adversity and the next 'big dry': Older farmers' mental health
J. D. Polain, H. L. Berry, J. O. Hoskin
Level: 2, State: Excluded

RefID: 1763, Suicide in a poor rural community in the Western Cape, South Africa: experiences of five suicide attempters and their families
Z. Holtman, S. Shelmerdine, L. London, A. Flisher
Level: 2, State: Excluded

RefID: 1780, Identifying Psychosocial Stressors of Well-Being and Factors Related to Substance Use Among Latino Day Laborers
N. J. Negi
Level: 2, State: Excluded

RefID: 1798, Suicide in Australian pesticide-exposed workers
E. MacFarlane, P. Simpson, G. Benke, M. R. Sim
Level: 2, State: Excluded

RefID: 1863, Social capital and self-rated health in Colombia: The good, the bad and the ugly
D. Hurtado, I. Kawachi, J. Sudarsky
Level: 2, State: Excluded

RefID: 1899, Acculturative stress as a risk factor of depression and anxiety in the Latin American immigrant population

H. W. Revollo, A. Qureshi, F. Collazos, S. Valero, M. Casas
Level: 2, State: Excluded

RefID: 1953, Maternal Predictors of Behavioral Problems Among Mexican Migrant Farmworker Children
M. L. D. Siantz, N. Coronado, T. Dovydaitis
Level: 2, State: Excluded

RefID: 1954, Medically reported work-related ill-health in the UK agricultural sector
S. J. Stocks, S. Turner, M. Carder, L. Hussey, R. McNamee, R. M. Agius
Level: 2, State: Excluded

RefID: 1960, Mental Health in Immigrants From Nontraditional Receiving Sites
L. Kiang, J. G. Grzywacz, A. J. Marin, T. A. Arcury, S. A. Quandt
Level: 2, State: Excluded

RefID: 1963, Job Demands and Pesticide Exposure Among Immigrant Latino Farmworkers
J. G. Grzywacz, S. A. Quandt, Q. M. Vallejos, L. E. Whalley, H. Y. Chen, S. Isom, D. B. Barr, T. A. Arcury
Level: 2, State: Excluded

RefID: 1974, Financial problems and psychological distress: Investigating reciprocal effects among business owners
M. J. Gorgievski, A. B. Bakker, W. B. Schaufeli, H. B. van der Veen, C. W. M. Giesen
Level: 2, State: Excluded

RefID: 1994, Day Laborers and Occupational Stress: Testing the Migrant Stress Inventory With a Latino Day Laborer Population
M. R. Duke, B. Bourdeau, J. D. Hovey
Level: 2, State: Excluded

RefID: 2016, Acculturative Stress, Depression, and Anxiety in Migrant Farmwork College Students of Mexican Heritage
O. L. Mejia, C. J. McCarthy
Level: 2, State: Excluded

RefID: 2048, Mood Disorders Hospitalizations, Suicide Attempts, and Suicide Mortality Among Agricultural Workers and Residents in an Area With Intensive Use of Pesticides in Brazil
A. Meyer, S. Koifman, R. J. Koifman, J. C. Moreira, J. D. Chrisman, Y. Abreu-Villaca
Level: 2, State: Excluded

RefID: 2098, Clinical leadership in rural psychiatry: Farmers' mental health and well-being
T. Robinson, H. Kurtz, B. Kelly, J. Fuller, L. Fragar, S. Roy, K. Deans, J. Croft, B. Hedger
Level: 2, State: Excluded

RefID: 2130, Stress and help-seeking for drought-stricken citrus growers in the Riverland of

South Australia
A. K. Staniford, M. F. Dollard, B. Guerin
Level: 2, State: Excluded

RefID: 2154, Measuring Job Characteristics and Mental Health Among Latino Farmworkers: Results from Cognitive Testing
J. G. Grzywacz, T. Alterman, C. Muntaner, S. Gabbard, J. Nakamoto, D. J. Carroll
Level: 2, State: Excluded

RefID: 2214, Children's behavioral traits and risk of injury: Analyses from a case-control study of agricultural households
K. F. Carlson, S. G. Gerberich, B. H. Alexander, A. S. Masten, T. R. Church, J. M. Shutske, A. D. Ryan, C. M. Renier
Level: 2, State: Excluded

RefID: 2217, The Psychosocial and Socioeconomic Consequences of Bovine Spongiform Encephalopathy (BSE): A Community Impact Study
D. Mitra, C. Amaratunga, R. Sutherns, V. Pletsch, W. Corneil, S. Crowe, D. Krewski
Level: 2, State: Excluded

RefID: 2237, Mental health of farmers in Southern Queensland: Issues and support
D. Hossain, R. Eley, J. Coutts, D. Gorman
Level: 2, State: Excluded

RefID: 2249, Water insecurity and emotional distress: Coping with supply, access, and seasonal variability of water in a Bolivian squatter settlement
A. Wutich, K. Ragsdale
Level: 2, State: Excluded

RefID: 2263, Improving mental health capacity in rural communities: Mental health first aid delivery in drought-affected rural New South Wales
G. M. Sartore, B. Kelly, H. J. Stain, J. Fuller, L. Fragar, A. Tonna
Level: 2, State: Excluded

RefID: 2274, Depressive symptoms in youth heads of household in Rwanda - Correlates and implications for intervention
N. W. Boris, L. A. Brown, T. R. Thurman, J. C. Rice, L. M. Snider, J. Ntaganira, L. N. Nyirazinyoye
Level: 2, State: Excluded

RefID: 2277, Farm Family Coping with Stress: The Impact of the 1998 Ice Storm
L. A. Sutherland, T. Glendinning
Level: 2, State: Excluded

RefID: 2299, Patterns of suicide by occupation in England and Wales: 2001-2005
H. Meltzer, C. Griffiths, A. Brock, C. Rooney, R. Jenkins

Level: 2, State: Excluded

RefID: 2302, Functioning in neck and low back pain from a 12-year perspective: A prospective population-based study
A. Thelin, S. Holmberg, N. Thelin
Level: 2, State: Excluded

RefID: 2315, The big dry: The link between rural masculinities and poor health outcomes for farming men
M. Alston, J. Kent
Level: 2, State: Excluded

RefID: 2323, Determinants of health in seasonal migrants: Coffee harvesters in Los santos, Costa Rica
R. L. Bolanos, T. Partanen, M. Berrocal, B. Alvarez, L. Cordoba
Level: 2, State: Excluded

RefID: 2344, Quality of life, work ability, and self employment: a population survey of entrepreneurs, farmers, and salary earners
S. I. Saarni, E. S. Saarni, H. Saarni
Level: 2, State: Excluded

RefID: 2362, Nerves as Embodied Metaphor in the Canada/Mexico Seasonal Agricultural Workers Program
A. Mysyk, M. England, J. A. A. Gallegos
Level: 2, State: Excluded

RefID: 2401, Anthropological and psychological merge: Design of a stress measure for Mexican farmworkers
S. A. Snipes, B. Thompson, K. O'Connor, R. Godina, G. Ibarra
Level: 2, State: Excluded

RefID: 2409, A sense of coherence and health. Salutogenesis in a societal context: Aland, a special case?
M. Eriksson, B. Lindstrom, J. Lilja
Level: 2, State: Excluded

RefID: 2425, Pesticide practices and suicide among farmers of the Sundarban region in India
A. N. Chowdhury, S. Banerjee, A. Brahma, M. G. Weiss
Level: 2, State: Excluded

RefID: 2446, Mortality among pesticide applicators exposed to chlorpyrifos in the agricultural health study
W. J. Lee, M. C. R. Alavania, J. A. Hoppin, J. A. Rusiecki, F. Kamel, A. Blair, D. P. Sandler
Level: 2, State: Excluded

RefID: 2517, Depressive symptoms normal elderly and associated factors in a cognitively population: the Tajiri Project
H. Ambo, K. Meguro, J. Ishizaki, M. Shimada, S. Yamaguchi, Y. Sekita, A. Yamadori
Level: 2, State: Excluded

RefID: 2520, Acute health effects of organophosphorus pesticides on Tanzanian small-scale coffee growers
A. V. F. Ngowi, D. N. Maeda, T. J. Partanen, M. P. Sanga, G. Mbise
Level: 2, State: Excluded

RefID: 2571, Relationship between diagnostic subtypes of depression and occupation in Japan
K. Otsuka, S. Kato
Level: 2, State: Excluded

RefID: 2588, The impact of urbanization on physical, physiological and mental health of Africans in the North West Province of South Africa: the THUSA study
H. H. Vorster, M. P. Wissing, C. S. Venter, H. S. Kruger, A. Kruger, N. T. Malan, J. H. de Ridder, F. J. Veldman, H. S. Steyn, B. M. Margetts, U. MacIntyre
Level: 2, State: Excluded

RefID: 2641, The coping inventory for stressful situations: Factorial structure and associations with personality traits and psychological health
R. Cosway, N. S. Endler, A. J. Sadler, I. J. Deary
Level: 2, State: Excluded

RefID: 2675, Pesticide-related health problems and diseases among farmers in the United Arab Emirates
M. M. M. Beshwari, A. Bener, A. Ameen, A. M. Al-Mehdi, H. Z. Ouda, M. A. H. Pasha
Level: 2, State: Excluded

RefID: 2695, Health and safety practices among farmers and other workers: a needs assessment
A. Hope, C. Kelleher, L. Holmes, T. Hennessy
Level: 2, State: Excluded

RefID: 2746, Suicide mortality and pesticide use among Canadian farmers
W. Pickett, W. D. King, R. E. M. Lees, M. Bienefeld, H. I. Morrison, R. J. Brison
Level: 2, State: Excluded

RefID: 2749, Methods used for suicide by farmers in England and Wales - The contribution of availability and its relevance to prevention
K. Hawton, J. Fagg, S. Simkin, L. Harriss, A. Malmberg
Level: 2, State: Excluded

RefID: 2798, Rural-urban women's experience of symptoms of depression related to economic hardship
B. J. Craft, D. R. Johnson, S. T. Ortega

Level: 2, State: Excluded

RefID: 2888, Increased risk of suicide with exposure to pesticides in an intensive agricultural area. A 12-year retrospective study
T. Parron, A. F. Hernandez, E. Villanueva
Level: 2, State: Excluded

RefID: 2897, Health and mental health among Mexican American migrants: Implications for survey research
R. D. Baer
Level: 2, State: Excluded

RefID: 2913, The impact of the environment on physical and mental health
A. G. Gallagher, K. Tierney
Level: 2, State: Excluded

RefID: 2924, Psychological distress and size of place: The epidemiology of rural economic stress
D. R. Hoyt, D. Odonnell, K. Y. Mack
Level: 2, State: Excluded

RefID: 3078, Major Depression in a Nonclinical Sample - Demographic and Clinical Risk-Factors for 1st Onset
W. Coryell, J. Endicott, M. Keller
Level: 2, State: Excluded

RefID: 3161, Mental-Health of North-Dakota Farm and Ranch Women - What Makes a Difference
H. Light, D. Hertsgaard, R. Hanson
Level: 2, State: Excluded

RefID: 3194, Differential Depression Effects on Families of Laborers, Farmers, and the Business Class a Survey of an Iowa Town
L. M. Conard
Level: 2, State: Excluded

RefID: 3248, “The Masks We Wear”: A Qualitative Study of Suicide in Australian Farmers
Lisa Kunde, Brian Kelly, Diego de Leo, Kõlves Kairi, Prasuna Reddy
Level: 2, State: Excluded

RefID: 3249, Health and Well-Being Among Young People From Canadian Farms: Associations With a Culture of Risk-Taking
William Pickett, Barbara Marlenga, Richard L. Berg
Level: 2, State: Excluded

RefID: 3392, Farm exit intention and wellbeing: A study of Australian farmers
Dominic Peel, Helen L. Berry, Jacki Schirmer

Level: 2, State: Excluded

RefID: 3452, Development of the Seasonal Migrant Agricultural Worker Stress Scale in Sanliurfa, Southeast Turkey
Zeynep Simsek, Fatma Ersin, Evin Kirmizitoprak
Level: 2, State: Excluded

RefID: 3475, An Investigation into the Human Element of On-farm Animal Welfare Incidents in Ireland
Catherine Devitt, Patricia Kelly, Martin Blake, Alison Hanlon, Simon J. More
Level: 2, State: Excluded

RefID: 3541, Help-seeking among Male Farmers: Connecting Masculinities and Mental Health
Philippe Roy, Gilles Tremblay, Steven Robertson
Level: 2, State: Excluded

RefID: 3565, Worker welfare on Kenyan export vegetable farms
Christoph R. Ehlert, MithÃ¶fer Dagmar, Hermann Waibel
Level: 2, State: Excluded

RefID: 3578, Economies, ethics and emotions: Farmer distress within the moral economy of agribusiness
Lia Bryant, Bridget Garnham
Level: 2, State: Excluded

RefID: 3621, Beyond discourses of drought: The micro-politics of the wine industry and farmer distress
Lia Bryant, Bridget Garnham
Level: 2, State: Excluded

RefID: 3664, The social experience of drought in rural Iran
Marzieh Keshavarz, Ezatollah Karami, Frank Vanclay
Level: 2, State: Excluded

RefID: 3684, Cardiovascular risk factors and psychological distress in Australian farming communities
Susan Brumby, Ananda Chandrasekara, Scott McCoombe, Peter Kremer, Paul Lewandowski
Level: 2, State: Excluded

RefID: 3731, The Hidden Health Burden of Environmental Degradation: Disease Comorbidities and Dryland Salinity
Peter C. Speldewinde, Angus Cook, Peter Davies, Philip Weinstein
Level: 2, State: Excluded

RefID: 3798, Psychosocial work environment among employed Swedish dairy and pig farmworkers

Christina Kolstrup, Peter Lundqvist, Stefan Pinzke
Level: 2, State: Excluded

RefID: 3822, A note on the effect of farmer mental health on adoption: The case of agri-environment schemes
B. Hounsome, R. T. Edwards, G. Edwards-Jones
Level: 2, State: Excluded

RefID: 3851, Stress among migrant and seasonal farmworkers in rural southeast North Carolina
Y. S. Kim-Godwin, G. A. Bechtel
Level: 2, State: Excluded

RefID: 3899, The Farm Partners program: addressing the problem of occupational stress in agriculture
J. J. May
Level: 2, State: Excluded

RefID: 4026, Variation in subjective well-being among black migrant farm workers in New York
P. S. K. Chi
Level: 2, State: Excluded

RefID: 4302, The Mental Health of the Organic Farmer: Psychosocial and Contextual Actors
C. Brigance, F. Soto Mas, V. Sanchez, A. J. Handal
Level: 2, State: Excluded

RefID: 4309, Midwestern Farm Widows: Adaptation Following Spousal Loss
J. W. Steinberg, G. M. Roux
Level: 2, State: Excluded

RefID: 4416, Are work demands associated with mental distress? Evidence from women in rural India
R. A. Richardson, A. Nandi, S. Jaswal, S. Harper
Level: 2, State: Excluded

RefID: 4478, A comparison of barriers to mental health support-seeking among farming and non-farming adults in rural South Australia
M. J. Hull, K. M. Fennell, K. Vallury, M. Jones, J. Dollman
Level: 2, State: Excluded

RefID: 4506, Trends and Characteristics of Occupational Suicide and Homicide in Farmers and Agriculture Workers, 1992-2010
W. Ringgenberg, C. Peek-Asa, K. Donham, M. Ramirez
Level: 2, State: Excluded

RefID: 4564, Improving Health Care for Spanish-Speaking Rural Dairy Farm Workers
C. Buckheit, D. Pineros, A. Olson, D. Johnson, S. Genereaux

Level: 2, State: Excluded

RefID: 4638, Health Needs Assessment of Plain Populations in Lancaster County, Pennsylvania
K. Miller, B. Yost, C. Abbott, S. Thompson, E. Dlugi, Z. Adams, M. Schulman, N. Strauss
Level: 2, State: Excluded

RefID: 4642, Farm-Related Concerns and Mental Health Status Among Norwegian Farmers
B. Logstein
Level: 2, State: Excluded

RefID: 4702, Predictors of mental complaints among Norwegian male farmers
B. Logstein
Level: 2, State: Excluded

RefID: 4729, Farmers' perceptions of health in the Riverland region of South Australia: 'If it's broke, fix it'
T. A. Rawolle, D. Sadauskas, G. van Kessel, J. Dollman
Level: 2, State: Excluded

RefID: 4752, Development and validation of a work stressor scale for Australian farming families
C. J. McShane, F. Quirk, A. Swinbourne
Level: 2, State: Excluded

RefID: 4784, Pesticide poisoning and neurobehavioral function among farm workers in Jiangsu, People's Republic of China
X. Zhang, M. Wu, H. Yao, Y. Yang, M. Cui, Z. Tu, L. Stallones, H. Xiang
Level: 2, State: Excluded

RefID: 4804, Differences in characteristics between suicide cases of farm managers compared to those of farm labourers in Queensland, Australia
U. Arnautovska, S. McPhedran, D. De Leo
Level: 2, State: Excluded

RefID: 4808, Mental and Physical Symptoms of Female Rural Workers: Relation between Household and Rural Work
M. R. Cezar-Vaz, C. A. Bonow, M. R. da Silva
Level: 2, State: Excluded

RefID: 4951, Work Ability Index (WAI) and its health-related determinants among Iranian farmers working in small farm enterprises
A. Rostamabadi, A. Mazloumi, A. Rahimi Foroushani
Level: 2, State: Excluded

RefID: 5011, Occupational exposure to pesticides, nicotine and minor psychiatric disorders among tobacco farmers in southern Brazil

N. M. Faria, A. G. Fassa, R. D. Meucci, N. S. Fiori, V. I. Miranda
Level: 2, State: Excluded

RefID: 5024, Veterinarian challenges to providing a multi-agency response to farm animal welfare problems in Ireland: responding to the human factor
C. Devitt, P. Kelly, M. Blake, A. Hanlon, S. J. More
Level: 2, State: Excluded

RefID: 5208, Empirical evidence suggests adverse climate events have not affected Australian women's health and well-being
J. R. Powers, D. Loxton, J. Baker, J. L. Rich, A. J. Dobson
Level: 2, State: Excluded

RefID: 5218, Mental health in Alberta grain farmers using pesticides over many years
N. Cherry, I. Burstyn, J. Beach, A. Senthilselvan
Level: 2, State: Excluded

RefID: 5233, Health status and needs of Latino dairy farmworkers in Vermont
D. Baker, D. Chappelle
Level: 2, State: Excluded

RefID: 5331, Reducing psychological distress and obesity in Australian farmers by promoting physical activity
S. Brumby, A. Chandrasekara, S. McCoombe, S. Torres, P. Kremer, P. Lewandowski
Level: 2, State: Excluded

RefID: 5346, Farming fit? Dispelling the Australian agrarian myth
S. Brumby, A. Chandrasekara, S. McCoombe, P. Kremer, P. Lewandowski
Level: 2, State: Excluded

RefID: 5444, Distress among rural residents: does employment and occupation make a difference?
L. Fragar, H. J. Stain, D. Perkins, B. Kelly, J. Fuller, C. Coleman, T. J. Lewin, J. M. Wilson
Level: 2, State: Excluded

RefID: 5492, The potential impact on farmer health of enhanced export horticultural trade between the U.K. and Uganda
P. Cross, R. T. Edwards, P. Nyeko, G. Edwards-Jones
Level: 2, State: Excluded

RefID: 5499, Challenges of conducting a large rural prospective population-based cohort study: the Keokuk County Rural Health Study
A. M. Stromquist, J. A. Merchant, C. Zwerling, L. F. Burmeister, W. T. Sanderson, K. M. Kelly
Level: 2, State: Excluded

RefID: 5514, Chronic back pain and associated work and non-work variables among

farmworkers from Starr County, Texas
E. M. Shipp, S. P. Cooper, D. J. del Junco, G. L. Delclos, K. D. Burau, S. Tortolero, R. E. Whitworth
Level: 2, State: Excluded

RefID: 5577, Mental health status among rural women of reproductive age: findings from the Central Pennsylvania Women's Health Study
M. M. Hillemeier, C. S. Weisman, G. A. Chase, A. M. Dyer
Level: 2, State: Excluded

RefID: 5579, Social networks and mental health among a farming population
H. J. Stain, B. Kelly, T. J. Lewin, N. Higginbotham, J. R. Beard, F. Hourihan
Level: 2, State: Excluded

RefID: 5606, Health and safety needs of older farmers: part I. Work habits and health status
S. K. Lizer, R. E. Petrea
Level: 2, State: Excluded

RefID: 5761, Depressive symptoms in adolescents living in rural America
A. R. Peden, D. B. Reed, M. K. Rayens
Level: 2, State: Excluded

RefID: 5770, Vacations improve mental health among rural women: the Wisconsin Rural Women's Health Study
V. Chikani, D. Reding, P. Gunderson, C. A. McCarty
Level: 2, State: Excluded

RefID: 5802, The impact of depression is unevenly distributed in the population
D. Isacson, K. Bingefors, L. von Knorring
Level: 2, State: Excluded

RefID: 5819, Gunshot suicides in England--a multicentre study based on coroners' records
L. Sutton, K. Hawton, S. Simkin, P. Turnbull, N. Kapur, O. Bennewith, D. Gunnell
Level: 2, State: Excluded

RefID: 5834, Impact of a foot and mouth disease crisis on post-traumatic stress symptoms in farmers
M. Olff, M. W. Koeter, E. H. Van Haaften, P. H. Kersten, B. P. Gersons
Level: 2, State: Excluded

RefID: 5917, Safety practices, neurological symptoms, and pesticide poisoning
C. Beseler, L. Stallones
Level: 2, State: Excluded

RefID: 5968, Pesticide illness, farm practices, and neurological symptoms among farm residents in Colorado

L. Stallones, C. Beseler
Level: 2, State: Excluded

RefID: 5977, A comparison of cardiovascular disease risk factors in farm and non-farm residents: the Wisconsin Rural Women's Health Study
C. McCarty, P. H. Chyou, L. Ziegelbauer, D. Kempf, D. McCarty, P. Gunderson, D. Reding
Level: 2, State: Excluded

RefID: 6036, Pesticide use and pesticide-related symptoms among black farmers in the Agricultural Health Study
S. A. Martin, D. P. Sandler, S. D. Harlow, D. L. Shore, A. S. Rowland, M. C. Alavanja
Level: 2, State: Excluded

RefID: 6582, Relationship of hardiness and current life events to perceived health in rural adults
H. J. Lee
Level: 2, State: Excluded

RefID: 6630, Family response to the farm crisis: a study in coping
M. Van Hook
Level: 2, State: Excluded

RefID: 6686, The farm crisis: an analysis of social psychological distress among North Carolina farm operators
M. D. Schulman, P. S. Armstrong
Level: 2, State: Excluded

Author	Title
J. C. Sandberg, H. T. Nguyen, S. A. Quandt, H. Y. Chen, P. Summers, F. O. Walker,	Sleep Quality Among Lati
A. G. Tribble, P. Summers, H. Y. Chen, S. A. Quandt, T. A. Arcury	Musculoskeletal pain, de
D. C. Mora, S. A. Quandt, H. Y. Chen, T. A. Arcury	Associations of Poor Hou
J. C. Sandberg, J. W. Talton, S. A. Quandt, H. Y. Chen, M. Weir, W. R. Doumani, A.	Association Between Hou
K. O'Connor, M. Stoecklin-Marois, M. B. Schenker	Examining nervios among
H. Park, N. L. Sprince, M. Q. Lewis, L. F. Burmeister, P. S. Whitten, C. Zwerling	Risk factors for work-rela
B. H. Chaney, E. Torres	Covariates of Identified S
A. K. Ramos, D. J. Su, L. Lander, R. Rivera	Stress Factors Contributi
J. G. Grzywacz, T. Alterman, S. Gabbard, R. Shen, J. Nakamoto, D. J. Carroll, C. Mu	Job Control, Psychologica
E. Alderete, W. A. Vega, B. Kolody, S. Aguilar-Gaxiola	Depressive symptomatol
J. G. Linn, B. A. Husaini	Determinants of Psycholo
M. J. Belyea, L. M. Lobao	Psychosocial consequenc
M. K. Rayens, D. B. Reed	Predictors of depressive s
C. L. Beseler, L. Stallones	Structural equation mode
C. L. Beseler, L. Stallones	Safety knowledge, safety
C. L. Beseler, L. Stallones	A cohort study of pesticid
C. L. Beseler, L. Stallones	Structural equation mode
J. A. Merchant, A. M. Stromquist, K. M. Kelly, C. Zwerling, S. J. Reynolds, L. F. Bur	Chronic disease and injur
H. Xiang, L. Stallones, T. J. Keefe	Back pain and agricultura
C. A. Pulgar, G. Trejo, C. Suerken, E. H. Ip, T. A. Arcury, S. A. Quandt	Economic Hardship and D
Rebecca Crain, Joseph G. Grzywacz, Melody Schwantes, Scott Isom, Sara A. Quan	Correlates of Mental Hea
Ann E. Hiott, Joseph G. Grzywacz, Stephen W. Davis, Sara A. Quandt, Thomas A. A	Migrant Farmworker Stre
R. D. Scarth, L. Stallones, C. Zwerling, L. F. Burmeister	The prevalence of depres
Y. S. Kim-Godwin, M. O. Maume, J. A. Fox	Depression, Stress, and In
H. T. Nguyen, S. A. Quandt, J. G. Grzywacz, H. Y. Chen, L. Galvan, M. H. Kitner-Tri	Stress and cognitive func
J. C. Sandberg, J. G. Grzywacz, J. W. Talton, S. A. Quandt, H. Y. Chen, A. B. Chatte	A Cross-Sectional Explora
J. G. Grzywacz, A. B. Chatterjee, S. A. Quandt, J. W. Talton, H. Y. Chen, M. Weir, T	Depressive Symptoms an
J. G. Grzywacz, S. A. Quandt, H. Y. Chen, S. Isom, L. Kiang, Q. Vallejos, T. A. Arcury	Depressive Symptoms Am
R. D. Scarth, C. Zwerling, M. Q. Lewis, L. F. Burmeister	Depression and risk facto
A. K. Ramos, G. Carlo, K. Grant, N. Trinidad, A. Correa	Stress, Depression, and O
D. B. Reed, M. K. Rayens, C. K. Conley, S. Westneat, S. M. Adkins	Farm elders define health
J. D. Hovey, C. G. Magana	Exploring the mental hea
L. Stallones, C. Beseler	Pesticide poisoning and d
M. I. Z. Roblyer, J. G. Grzywacz, C. K. Suerken, G. Trejo, E. H. Ip, T. A. Arcury, S. A.	Interpersonal and social c
K. M. Khan, R. Baidya, A. Aryal, J. R. Farmer, J. Valliant	Neurological and mental
M. Schwantes, C. McKinney, N. Hannibal	Music therapy's effects o
A. M. Bush, S. Westneat, S. R. Browning, J. Swanberg	Missed Work Due to Occu
J. G. Grzywacz, T. Alterman, C. Muntaner, R. Shen, J. Li, S. Gabbard, J. Nakamoto,	Mental Health Research v
T. A. Arcury, G. Trejo, C. K. Suerken, J. G. Grzywacz, E. H. Ip, S. A. Quandt	Work and Health Among
J. D. Hovey, G. Hurtado, L. D. Seligman	Findings for a CBT Suppor
S. R. Terrazas, A. McCormick	Coping Strategies That M

Journal	Country	Study populat	Gender	Sampling	Study design
Journal of Immigrant and Minority	USA	Migrant farmw	Men	purposive sam	Cross-sectiona
Archives of Environmental & Occup	USA	Migrant farmw	Men	convenience s	Prospective co
Journal of Agromedicine	USA	Migrant farmw	Men	convenience s	Cross-sectiona
Journal of Immigrant and Minority	USA	Migrant farmw	Men	convenience s	Cross-sectiona
J Immigr Minor Health	USA	Migrant farmw	Men	Stratified rand	Prospective co
J Occup Environ Med	USA	Farmers	Men	Stratified rand	Prospective co
International Journal of Environme	USA	Migrant farmw	Mixed	convenience s	Cross-sectiona
Journal of Immigrant and Minority	USA	Migrant farmw	Mixed	convenience s	Cross-sectiona
Journal of Occupational and Enviro	USA	Migrant farmw	Mixed	multi-stage sa	Cross-sectiona
Journal of Community Psychology	USA	Migrant farmw	Mixed	multi-stage sa	Cross-sectiona
Journal of Community Psychology	USA	Farmers	Mixed	Simple-randor	Cross-sectiona
Rural sociology	USA	Farmers	Mixed	Stratified rand	Cross-sectiona
J Rural Health	USA	Farmers	Mixed	convenience s	Cross-sectiona
J Agromedicine	USA	Farmers	Mixed	Stratified rand	Cross-sectiona
Am J Ind Med	USA	Farmers	Mixed	Stratified rand	Cohort
Ann Epidemiol	USA	Farmers	Mixed	Stratified rand	Prospective co
J Agromedicine	USA	Farmers	Mixed	purposive sam	Cross-sectiona
J Rural Health	USA	Farmers	Mixed	Stratified rand	Cross-sectiona
Am J Ind Med	USA	Farmers	Mixed	Stratified rand	Cross-sectiona
J Immigr Minor Health	USA	Migrant farmw	Women	purposive sam	Cross-sectiona
Journal of rural health	USA	Migrant farmw	Men	convenience s	RCT
Journal of rural health	USA	Migrant farmw	Men	convenience s	Cross-sectiona
Am J Ind Med	USA	Farmers	Men	Stratified rand	Cross-sectiona
Journal of Immigrant and Minority	USA	Migrant farmw	Mixed	convenience s	Cross-sectiona
American Journal of Industrial Med	USA	Migrant farmw	Mixed	convenience s	Prospective co
Journal of Agromedicine	USA	Migrant farmw	Mixed	Stratified rand	Cross-sectiona
Journal of Agromedicine	USA	Migrant farmw	Mixed	convenience s	Cross-sectiona
Cultural Diversity & Ethnic Minority	USA	Migrant farmw	Mixed	convenience s	Cross-sectiona
Journal of agromedicine	USA	Farmers	Mixed	Stratified rand	Cross-sectiona
Safety (Basel)	USA	Migrant farmw	Mixed	convenience s	Cross-sectiona
Workplace Health Saf	USA	Farmers	Mixed	convenience s	Cross-sectiona
J Psychol	USA	Migrant farmw	Mixed	Not reported	Cross-sectiona
Ann Epidemiol	USA	Farmers	Mixed	multi-stage sa	Cross-sectiona
Women & Health	USA	Migrant farmw	Women	purposive sam	Cross-sectiona
Annals of Agricultural and Environm	USA	Farmers	Men	Simple-randor	Cross-sectiona
Arts in Psychotherapy	USA	Migrant farmw	Men	convenience s	RCT
Journal of Agricultural Safety and H	USA	Migrant farmw	Mixed	purposive sam	Cross-sectiona
Journal of immigrant and minority	USA	Migrant farmw	Mixed	multi-stage sa	Cross-sectiona
Journal of Occupational and Enviro	USA	Migrant farmw	Women	convenience s	Cross-sectiona
Current Psychology	USA	Migrant farmw	Women	purposive sam	Non-Randomi
Hispanic Journal of Behavioral Scier	USA	Migrant farmw	Mixed	convenience s	Cross-sectiona

Measurement	Scale	Outcome
Self-report	CES-D	Binary
Self-report	CES-D	Binary
Self-report	CES-D	Binary
Self-report	CES-D	Binary
Self-report	CES-D	Binary
Self-report	CES-D	Binary
Self-report	CES-D	Binary
Self-report	CES-D	Binary
Self-report	CES-D	Binary
Self-report	CES-D	Binary
Self-report	CES-D	Binary
Self-report	CES-D	Binary
Self-report	CES-D	Binary
Self-report	CES-D	Binary
Self-report	CES-D	Binary
Self-report	CES-D	Binary
Self-report	CES-D	Binary
Self-report	CES-D	Binary
Self-report	CES-D	Binary
Self-report	CES-D	Binary
Self-report	CES-D	Binary,Continuous
Self-report	CES-D	Binary,Continuous
Self-report	CES-D	Binary,Continuous
Self-report	CES-D	Binary,Continuous
Self-report	CES-D	Binary,Continuous
Self-report	CES-D	Binary,Continuous
Self-report	CES-D	Binary,Continuous
Self-report	CES-D	Binary,Continuous
Self-report	CES-D	Binary,Continuous
Self-report	CES-D	Binary,Continuous
Self-report	CES-D	Binary,Continuous
Self-report	CES-D	Binary,Continuous
Self-report	CES-D	Binary,Continuous
Self-report	CES-D	Binary,Continuous
Self-report	CES-D	Continuous
Self-report	CES-D	Continuous
Self-report	CES-D	Continuous
Self-report	CES-D	Continuous
Self-report	CES-D	Continuous
Self-report	CES-D	Continuous
Self-report	CESD-R	Binary
